# *Fragaria × ananassa* cv. Senga Sengana Leaf: An Agricultural Waste with Antiglycation Potential and High Content of Ellagitannins, Flavonols, and 2-Pyrone-4,6-dicarboxylic Acid

**DOI:** 10.3390/molecules27165293

**Published:** 2022-08-19

**Authors:** Izabela Fecka, Katarzyna Bednarska, Maciej Włodarczyk

**Affiliations:** Department of Pharmacognosy and Herbal Medicines, Faculty of Pharmacy, Wroclaw Medical University, Borowska 211a, 50-556 Wroclaw, Poland

**Keywords:** agrimoniin (CAS No: 82203-01-8), ellagitannins, flavonols, polyphenols, PDC (CAS No: 72698-24-9), miquelianin (CAS No: 22688-79-5), *Fragaria* × *ananassa*, methylglyoxal, glycation inhibitor

## Abstract

Strawberry leaves are considered a valuable waste material; so far, mainly due to their antioxidant properties. Since the annual production of this crop is high, our study aimed to thoroughly examine the chemical composition and antidiabetes-related bioactivity of *Fragaria* × *ananassa* leaf of its popular and productive cultivar Senga Sengana. Leaves from three different seasons, collected after fruiting, were extensively analyzed (UHPLC-qTOF-MS/MS, HPLC-DAD). Some individual components were isolated and quantified, including specific flavonol diglycosides (e.g., 3-*O*-[β-xylosyl(1‴→2″)]-β-glucuronosides). The separated quercetin glycosides were tested in an antiglycation assay, and their methylglyoxal uptake capacity was measured. In addition, the biodegradable polyester precursor 2-pyrone-4,6-dicarboxylic acid (PDC) was confirmed at relatively high levels, providing further opportunity for strawberry leaf utilization. We want to bring to the attention of the food, pharmaceutical, and cosmetic industries the Senga Sengana strawberry leaf as a new botanical raw material. It is rich in PDC, ellagitannins, and flavonols—potent glycation inhibitors.

## 1. Introduction

Plant polyphenols provided to the human body with herbs, vegetables, or fruits significantly influence the digestive system, internal organs, and tissue activity. However, their bio-accessibility is diverse and conditioned by their chemical structure. The available epidemiological and interventional studies featuring healthy volunteers and patients show that polyphenols with antioxidative, antiglycative, and antiphlogistic properties reduce the risk of some chronic noncommunicable diseases, such as diabetes, metabolic syndrome, fatty liver disease, cardiovascular disease, and some cancers. Undoubtedly, in the etiology of those illnesses, an important role is played by pathogenic oxidative and carbonyl stress and ongoing inflammation [1,2,3]. In this regard, much attention has been paid to the properties of commonly consumed polyphenols [4,5]. There is also ongoing research on the interactions between polyphenols and gut microbiota to elucidate their health benefits. The data on flavonoids and ellagitannins are of particular interest [6,7].

In traditional medicine, leaves of species providing berry fruits (such as blackberry, raspberry, or wild strawberry) were used to cure gastroenteritis and mild diarrhea, strengthen the heart and circulatory system, and in metabolic disturbances to ‘improve metabolism’ and for ‘blood purification.’ The effectiveness of those recommendations is not sufficiently documented; therefore, their therapeutic application is currently limited. For example, the wild strawberry leaf is included in several multi-component food supplements and traditional herbal medicines (traditional botanical drugs) in the EU, intended to relieve non-specific diarrhea or enhance diuresis and metabolism. Similarly, it is used as a component of notified and licensed products in the USA and Canada [8]. Herbal medicines containing wild strawberry leaf are usually available as herbal teas for oral use.

It should be noted that wild strawberry leaf is the common name shared by the dried leaves of at least four species: *Fragaria vesca* L., *F. moschata* Weston, *F. viridis* Weston, and *F. ananassa* (Weston) Duch. ex Rozier. This raw plant material is described, inter alia, in the European Union herbal monograph *Fragariae folium* (EMA/HMPC/432278/2015). Leaves of *Fragaria* species with or without petioles can be harvested throughout the growing season. However, according to older data, wild strawberry leaf for therapeutic purposes was collected during the flowering period [8].

Phytochemical studies have shown a similar chemical profile of leaves from *Fragaria* species [9]. Nevertheless, scientific references focus mainly on *F. vesca* (woodland strawberry). The woodland strawberry leaf contains up to 12% tannins (ellagitannins and proanthocyanidins), 0.2–4% flavonoids (glycosides of flavonols), phenolic acids (ellagic acid, gallic acid, hydroxycinnamic acids), triterpenes, traces of essential oil, and others [10]. Its main components are quercetin, kaempferol glycosides, catechin, and agrimoniin [8,11]. The chemical identity of many others has not been revealed yet.

Due to the efforts of growers and the adaptability of *F. ananassa* plants, strawberry cultivation has spread almost worldwide. According to FAO data, world strawberry production in 2020 was nearly 7 million tonnes, and the top three global strawberry producers were China, the USA, and Egypt [12]. The Senga Sengana strawberry has been a leading industrial cultivar for many years. It is characterized by abundant foliage, consisting of large, shiny leaves with a dark green surface. The older leaves are removed in the crops about 2–3 weeks after harvesting the berries by pruning all the plants (July/August). The treatment is applied to 2- and 3-year-old strawberries. The plant material remaining after this procedure has unexplored potential for utility.

For these reasons, in the present study we performed a detailed examination of the chemical composition of *F. ananassa* cv. Senga Sengana leaves gathered after fruiting as regards polyphenols (including tannins, flavonoids, phenolic acids) and carboxylic acids. The results obtained for Senga Sengana leaves were compared to leaves of *F. vesca*, a research model used in other studies. Since flavonoids are characterized by their ability to take up methylglyoxal and inhibit the destructive glycation of bio-molecules [13], we decided to compare the effects of isolated quercetin glycosides with known glycation inhibitors such as aminoguanidine and metformin. Based on this, we identified a new botanical raw material with valuable phytochemicals and high utility potential.

## 2. Results

The leaves of species from the *Fragaria* genus are considered rich in antioxidants and thus of interest to the food, pharmaceutical, and cosmetic industries. Among them, garden strawberry leaves are the most broadly available renewable resource that can be harvested yearly after fruiting. There are many cultivars of garden strawberry; however, we do not know the complete chemical composition of most of them—neither the fruit nor the leaf.

The Senga Sengana strawberry has been consistently touted as a leading industrial cultivar for many years. It was first bred in Germany, and today it performs widely in commercial and amateur cultivation.

The examined leaves of *F. ananassa* cv. Senga Sengana were collected in July of three different years from a local plantation (FaSS1, FaSS2) and the experimental farm COBORU (FaSS3). Preliminary chromatographic tests of 50% water-methanol extracts of them revealed a significant content of polar polyphenolic metabolites. Among other components, the presence of numerous flavonoids and hydrolyzable tannins was confirmed. However, we were unable to confirm the identity of several peaks with certainty, and this became one of the targets of the present experiment. Therefore, the dried leaves of FaSS1 were intended to isolate tannins, flavonoids, and derivatives of phenolic and carboxylic acids, whereas FaSS3 leaves were used for final UHPLC-qTOF-MS/MS profiling (Table 1). Since the diploid woodland strawberry (*F. vesca*) is frequently used to study the more complex octoploid garden strawberry (*F. ananassa*), we compared the leaf chemistry of the Senga Sengana strawberry with two woodland strawberry reference samples (Fv1, Fv2).

In-depth investigation of *F. ananassa* cv. Senga Sengana leaf polyphenols was carried out using various chromatographic (CC, TLC, HPLC-DAD, UHPLC-qTOF-MS/MS) and spectroscopic methods: ultraviolet to visible light spectroscopy (UV-VIS), electrospray ionization mass spectrometry (ESI-qTOF-MS), as well as one-dimensional (^1^H-, ^13^C-NMR, DEPT) and two-dimensional nuclear magnetic resonance spectroscopy (COSY, NOESY, HSQC, HMBC). In addition, plant extracts subjected to qualitative and quantitative analysis were prepared from leaves of both garden and woodland strawberries, using a procedure described previously [11].

A vital step to characterize the polyphenols of *F. ananassa* cv. Senga Sengana leaf was the separation of individual compounds on a preparative scale. Dried leaves of FaSS1 (500 g) were exhaustively extracted with water-acetone (1+1). The obtained aq. acetone extract (FaSS-A) was concentrated and fractionated using column chromatography on octadecyl, silica gel Si60 and Sephadex LH-20. From the octadecyl column, we obtained 13 fractions rich in polyphenols and other components (FaSS-0, FaSS-I to FaSS-XII), which were purified on silica gel Si60 (FaSS-I) or Sephadex LH-20 (FaSS-0, FaSS-II to FaSS-IX). FaSS-X and FaSS-XI, containing one dominant component each, were concentrated and allowed to crystallize from water-methanol (Figure S1). All isolated compounds were identified using authentic standards and tannins previously separated by the authors from various sources (if applicable; co-chromatography: TLC on silica gel 60 in mobile phases **E**, **F**, **P**—comparison of R*_f_*, color, and its intensity; HPLC-DAD—t*_R_*, UV-VIS spectra—*λ*_max_ and absorption profile) (Figures S2–S5), and characterized by ESI-qTOF-MS/MS in negative mode. In addition, 1D- and 2D-NMR experiments were used for structural elucidation of hydrolyzable tannins, flavonoid glycosides, and derivatives of phenolic and carboxylic acids separated from FaSS-A (see Appendix A and Appendix B for isolation/identification details and Supplementary Materials for MS/MS and NMR details).

To summarize, sixteen individual compounds were isolated from the aq. acetone extract of Senga Sengana leaves. These included four hydrolyzable tannins, compounds **3** (3-*O*-galloylquinic acid) [14,15], **5** (5*-O-*galloylquinic acid) [14,15], **7** (pedunculagin) [16], and **10** (agrimoniin) [17,18]; seven flavonoid glycosides, compounds **8** ((2*R*,3*R*)-taxifolin-3-*O*-β-glucoside) [19], **9** (quercetin-3-*O*-β-glucuronoside-7-*O*-β-glucoside) [20], **11** (quercetin-3-*O*-[β-xylosyl(1‴→2″)]-β-glucuronoside) [21], **12** (quercetin-3-*O*-β-glucuronoside) [22], **13** (kaempferol-3-*O*-[β-xylosyl(1‴→2″)]-β-glucuronoside, newly described), **14** (isorhamnetin-3-*O*-β-glucuronoside) [23], and **15** (tiliroside) [24]; and five other compounds: **1** (2-pyrone-4,6-dicarboxylic acid, PDC) [25], **2** (quinic acid), **4** (**4a**: 1-*O*-protocatechuoyl-β-xylose, newly described), **6** (catechin) [26], and **16** (ellagic acid).

**Figure 1:**
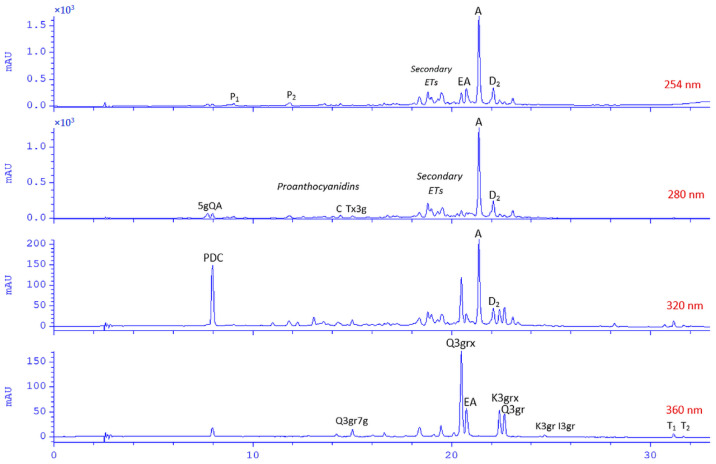
HPLC-DAD chromatograms of 50% water-methanol extract from leaves of *Fragaria × ananassa* cv. Senga Sengana (FaSS3, 1:200); λ given in red. Abbreviations: A, agrimoniin (**10**); C, catechin (**6**); D2, davuriciin D2 = fragariin A (not isolated); EA, ellagic acid (**16**); ETs, ellagitannins; 5gQA, 5-*O*-galloylquinic acid (**5**); I3gr, isorhamnetin-3-*O*-β-glucuronoside (**14**); K3gr, kaempferol-3-*O*-β-glucuronoside; K3grx, kaempferol-3-*O*-[β-xylosyl(1‴→2″)]-β-glucuronoside (**13**); P1 and P2, pedunculagin, α/β (**7**); PDC, 2-pyrone-4,6-dicarboxylic acid (**1**); Q3gr, quercetin-3-*O*-β-glucuronoside (miquelianin; **12**); Q3grx, quercetin-3-*O*-[β-xylosyl(1‴→2″)]-β-glucuronoside (flagarin; **11**); Q3gr7g, quercetin-3-*O*-β-glucuronoside-7-*O*-β-glucoside (**9**); Tx3g, (2*R*,3*R*)-taxifolin-3-*O*-β-glucoside (**8**); T1 and T2, tiliroside, trans/cis (**15**).

**Figure 2:**
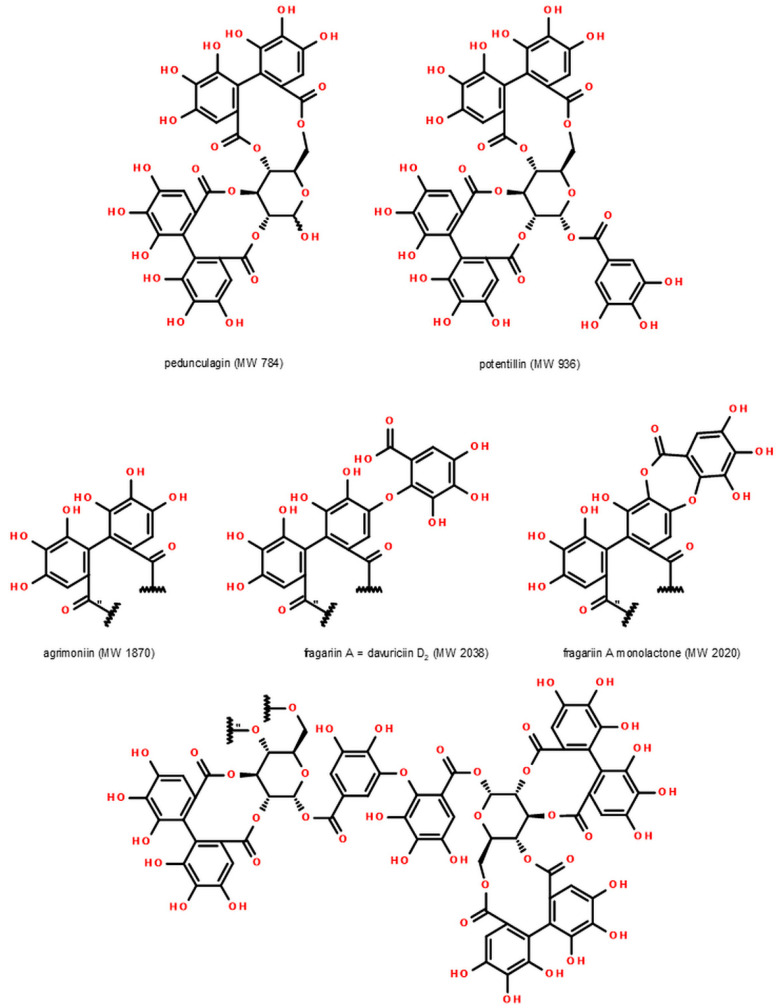
Ellagitannins described in the leaves of *Fragaria × ananassa* cv. Senga Sengana (including pedunculagin (**7**), and agrimoniin (**10**)).

**Figure 3:**
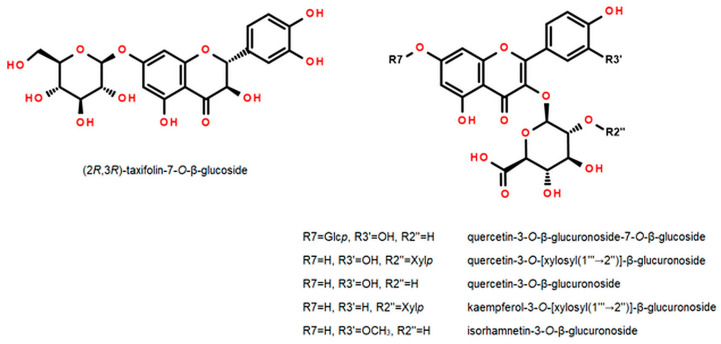
Flavonoids identified in the leaves of *Fragaria × ananassa* cv. Senga Sengana (compounds **8** (taxifolin-3-*O*-β-glucoside), **9** (quercetin-3-*O*-β-glucuronoside-7-*O*-β-glucoside), **11** (quercetin-3-*O*-[β-xylosyl(1‴→2″)]-β-glucuronoside), **12** (quercetin-3-*O*-β-glucuronoside), **13** (kaempferol-3-O-[β-xylosyl(1‴→2″)]-β-glucuronoside), **14** (isorhamnetin-3-*O*-β-glucuronoside).

**Figure 4:**
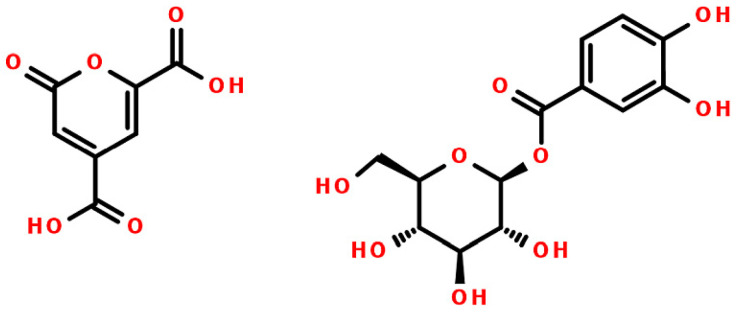
Other compounds identified in the leaves of *Fragaria × ananassa* cv. Senga Sengana: **1** (2-pyrone-4,6-dicarboxylic acid, PDC, **left**), and **4a**: 1-*O*-protocatechuoyl-β-xylose (**right**).

To identify minor Senga Sengana components, we performed UHPLC-qTOF-MS/MS profiling with co-chromatography (Table 1). Figure 1 shows a typical HPLC-DAD chromatogram of 50% water-methanol extract from leaves of *F. ananassa* cv. Senga Sengana. The structures of main ellagitannins identified in the analyzed leaves are presented in Figure 2, those of flavonoids in Figure 3, while other are depicted in Figure 4.

### 2.1. Hydrolyzable Tannins

Agrimoniin (**10**) was previously obtained from *Agrimonia pilosa* Ledeb. (syn. *A. japonica* (Miq.) Koidz.) and *Potentilla kleiniana* Wight et Arnott. [17,18,29], as well as from *F. ananassa* Duch. and *F. nipponica* Makino leaves [29], and berries of woodland and garden strawberry [45]. The content of agrimoniin in plant materials FaSS1 and FaSS2 was determined as ca. 4.4% and 3% (dry matter), respectively, while it was 8.1% in FaSS3 (Table 2). Pedunculagin (**7**) was previously reported in strawberry leaves by Okuda et al. [29].

Monogalloylquinic acids **3** and **5** (classified in gallotannins) were identified as 3- and 5-*O*-galloylquinic acid, respectively [14,15]. The content of dominant 5-*O*-galloylquinic acid in Senga Sengana leaves ranged from 0.4 to 0.6% of dry matter (Table 2). The other regioisomers of monogalloylquinic acid (Table 1) were present in much lower amounts.

The identity of other minor ellagitannins (ETs: davuriciin D_2_/fragariin A; laevigatins B/C/F; potentillin; galloyl-HHDP-glucose isomers such as sanguiin H-4; digalloyl-HHDP-glucose), as well as gallotannins (3,5-*O*-digalloylquinic acid; mono-, di- and trigalloylglucose) [27,32,34,35,36,37] in strawberry leaf extracts and fractions was determined using UHPLC-qTOF-MS/MS (Table 1). Davuriciin D_2_ was isolated for the first time from the root of *Rosa davurica* Pall. [37]. Ellagitannin with MW 2038 Da was also obtained from strawberry fruit pomace under the name fragariin A [32]. Analysis of the published spectroscopic data and structures proposed for these ETs by their authors indicates that they are most likely identical. In addition, four ETs with molecular weights of 934 (two isomers), 1236, and 2020 Da were detected in Senga Sengana leaves. The same compounds were observed in pseudo-fruits, flowers, and leaves of several other *F. ananassa* cultivars, but their formulas were not elucidated [27,28,30,36]. Fragmentation of those minor strawberry ETs in the negative ion mode provided products typical for HHDP esters (*m*/*z* 481 and 301) and an additional ion derived from a low molecular depside at *m*/*z* 451 (exactly 450.9950). The same signal has been observed on the MS^2^ spectrum of davuriciin D_2_/fragariin A (MW 2038 Da, *m*/*z* 1018.0749 [M−2H]^−2^), which is a valoneoyl analog of agrimoniin [37]. Therefore, the presence of the *m*/*z* 451 ion suggests a structural affinity, so we tentatively propose their chemical formulas starting with davuriciin D_2_/fragariin A (dimeric ellagitannin composed of α-pedunculagin and α-praecoxin A linked by DHDG, Figure 2). Ellagitannin-2020 is possibly a lactone of davuriciin D_2_/fragariin A (in the valoneoyl group) composed of α-pedunculagin and α-praecoxin D coupled by DHDG. The remaining compounds are secondary ellagitannins formed by the neutral loss of monomeric ellagitannin molecules, i.e., davuriciin D_2_/fragariin A lactone without the pedunculagin fragment (ET-1236 → 2020 − 784 = 1236) and davuriciin D_2_/fragariin A lactone without pedunculagin and DHDG coupler (ET-934 isomers → 2020 − 784 − 302 = 934). The latter compounds are probably α- and β-anomers of praecoxin D [31]. The absence of the DHDG lactone-derived ion in MS^2^ supports that thesis. As a result of the fragmentation of *m*-GOG type dimers and certain secondary ellagitannins, besides both ellagic acid (*m*/*z* 301) and decarboxylated monolactone of hexahydroxydiphenic acid (LHHDP−CO_2_, *m*/*z* 275) ions, a lactone of the dehydrodigallic acid ion (LDHDG, *m*/*z* 319) also occurred. Tannins with a valoneoyl group additionally released the diagnostically relevant ion of valoneic acid trilactone (VTL, *m*/*z* 451). The reported ETs with molecular weights below 1870 Da were probably products of partial degradation or metabolism of agrimoniin and davuriciin D_2_/fragariin A. D2 content was many times lower than agrimoniin at 0.2–0.8% of dry matter (Table 2). 

The quantitative analysis of ellagitannins in various parts of strawberry cultivars in relation to the development stage was the topic of work of Karlińska and co-workers [46].

### 2.2. Proanthocyanidins and Flavan-3-Ols

Condensed tannins were minor *F. ananassa* cv. Senga Sengana components. Several B-type proanthocyanidins (five dimers, three trimers, and one tetramer) were detected together with monomeric catechin by UHPLC-qTOF-MS/MS (*m*/*z* 289.0719, 577.1346, 865.1981, 1153.2610). Except for catechin (**6**), procyanidins B3 and C2, as well as oligomeric flavan-3-ol derivatives of both catechin and epicatechin, were recognized by UV-VIS profiles and MS fragmentations. Catechin and procyanidins B3 and B6 were formerly isolated from *F. ananassa* cv. Reikov roots [26].

### 2.3. Flavonoids

Seventeen flavonoid glycosides were recognized in extracts from Senga Sengana leaves (Table 1, Figure 3). Five compounds were derivatives of quercetin, five of kaempferol, one of isorhamnetin, and six were taxifolin glycosides. In this study, we separated the dominant flavonoids, especially those that have not been further characterized. Three diglycosides (**9**, **11**, and **13)**, as well as four monoglycosides (**8**, **12**, **14**, and **15**), were isolated from FaSS-A. Some of the noted flavonoids, including flavonol diglycosides (MW 594, 610, and 640 Da), were described previously in leaves of *F. ananassa* cv. Polka [27], in flowers of the cultivar Jonsok [28], and in pseudo-fruits of the Japanese cultivar Tochiotome [21]. Nevertheless, the abovementioned authors assigned the structures only tentatively by LC-MS.

The NMR data of flagarin (**11**) were in accordance with those published data [21]. The mentioned authors reported the presence of 3-*O*-β-(2″-Xyl*p*)-GlcA*p* of quercetin in strawberry fruits. However, the final semi-systematic name of this compound was given by them inversely in the sugar part, despite clear proof. Our results indicate that compound **11** should be quercetin-3-*O*-[β-xylosyl(1‴→2″)]-β-glucuronoside.

Compound **13** (yellow crystalline powder) had a pseudomolecular ion at *m*/*z* 593.1144 [M−H]^−^ (calc. 593.1148 for [C_26_H_25_O_16_]^−^), as well as a fragment ion at 285 [M−308−H]^−^ (aglycone). These data were relevant to hexuronoside-pentoside or rutinoside of kaempferol-like aglycone. Flavonoid **13** showed an analogic fragmentation pattern to **11**, so we deduced that sugars should be attached sequentially. After acid hydrolysis, **13** yielded glucuronic acid, xylose (mobile phase **S**), and kaempferol. ^13^C-NMR (DMSO-*d6*) data showed signals strictly corresponding to a glucuronosyl and a xylosyl, similarly as in **11**, but kaempferol was proved as an aglycone (doubled singlets of H-2′ & H-6′, and H-3′ & H-5′ in ring B). ^1^H-NMR showed precisely two doublets of anomeric protons (4.61 and 5.73 ppm), of which the shift with higher value was assigned to β-GlcA*p*. The glycosylation position was deduced similarly as in **11**, from C-3 upfield shift from ~135 to ~132 ppm together with downfield C-4 and C-1′ signals. The second sugar of glycone (β-Xyl*p*) was attached to the first one in the same way as in **11.** That was proved by 2D-NMR (NOESY: H-1‴↔H-2″) and downfield of the C-2″ signal of the β-GlcA*p* moiety (from ~73 to ~81 ppm). The NMR data were closely similar to **11**, an analogic glycoside of quercetin [21]. Our results indicate that compound **13** should be **3-*O*-[β-xylosyl(1‴→2″)]-β-glucuronoside of kaempferol**. This compound is newly described and supplied with NMR data (Appendix B, Supplementary Materials).

All spectroscopic data of **8** were clearly similar to those included in a previous paper [19] and different from those of Pan and Lundgren [47], but the spectrum revealed several overlapping isomer signals. That compound was also found in Tochiotome strawberries [21]. Other taxifolin pentosides and hexosides were also observed in LC-MS as minor components (Table 1). For example, taxifolin-3-*O*-α-arabinoside was isolated previously from roots of *F. ananassa* cv. Reikov [26].

Based on spectroscopic evidence, flavonoids **12, 14**, and **15** were identified as quercetin-3-*O*-β-glucuronoside (syn. Quercetin-3-*O*-β-glucuronide or miquelianin, *m*/*z* 477.0672), isorhamnetin-3-*O*-β-glucuronoside (3′-*O*-methyl-quercetin-3-*O*-β-glucuronoside, 491.0806), and tiliroside (kaempferol-3-*O*-β-(6″-*O*-*p*-coumaroyl) glucoside). Kaempferol, quercetin, and isorhamnetin-3-*O*-(6″-methyl) glucuronosides were formerly identified in fruits of *F. ananassa* cv. Falandi [48], whereas *cis*/*trans* tiliroside was identified in leaves of cv. Jonsok [42] and in pseudo-fruits of cultivars Tochiotome [41], Nohime [39], and Minomusume [40]. The structures of the most important flavonoids are presented in Figure 3.

The most abundant compound of the flavonoid fraction was quercetin-3-*O*-[β-xylosyl(1‴→2″)]-β-glucuronoside (**11**, flagarin ∼0.7%), followed by kaempferol-3-*O*-[β-xylosyl(1‴→2″)]-β-glucuronoside (**13**, ∼0.5%), quercetin-3-*O-*β-glucuronoside (**12**, 0.1–0.3%) and quercetin-3-*O*-β-glucuronoside-7-*O*-β-glucoside (**9**, ∼0.05%) (Table 2). The sum of flavonol glycosides in Senga Sengana leaves was determined at 1.5–1.7% (dry matter). Kårlund and coworkers quantified the quercetin and kaempferol glycosides in leaves of the strawberry cultivar Polka [27] at concentrations comparable to our study.

### 2.4. Phenolic and Carboxylic Acids

UHPLC-qTOF-MS/MS analysis of *F. ananassa* cv. Senga Sengana leaf extracts showed intense peaks of several phenolic acid derivatives, as well as carboxylic acids. Among them, **1** was identified as 2-pyrone-4,6-dicarboxylic acid (PDC, Figure 4). This heterocyclic dicarboxylic acid is considered as a chemotaxonomic marker of *Rosoideae* and was formerly reported in a concentration of 0.1–2% in the following genera: *Alchemilla*, *Agrimonia*, *Duchesnea*, *Filipendula*, *Fragaria*, *Geum*, *Potentilla*, *Rosa*, *Rubus*, *Sanguisorba*, and *Waldsteinia* [25]. PDC is also known as a low molecular product of catabolism (biodegradation) of plant polyphenols (phenolic acids, lignans, and lignin) by some microorganisms, e.g., by the soil bacterium *Sphingomonas paucimobilis* [49]. The biological origin of 2-pyran-4,6-dicarboxylic acid in strawberry leaves has not been definitively elucidated. In the examined raw plant material, the PDC content was in the range of 1–1.7% dry matter (Table 2). Carboxylic acid **2**, obtained from the same fraction as PDC, was identified as a cyclic polyol named quinic acid. The presence of citric acid was also confirmed in Senga Sengana leaves (Table 1).

Compound **4a** was separated as an off-white crystalline powder from fraction FaSS-I. Its pseudomolecular ion *m*/*z* at 285.0614 [M−H]^−^ (calc. 285.0616 for [C_12_H_13_O_8_]^−^) was relevant to *O*-pentoside of dihydroxybenzoic acid or dihydroxybenzoyl-pentose. Compound **4a** produced both radical aglycone (Y_0_−H)^−^^•^ and aglycone Y_0_^−^ ions at *m*/*z* 152 and 153 (by homolytic and heterolytic cleavage of the deprotonated precursor ion), as well as at 108 and 109 [50], corresponding to a dihydroxybenzoic acid residue after the loss of pentose followed by decarboxylation. The ions formed by the homolytic cleavage were dominant, which suggested the presence of two free phenolic groups in the benzene ring. In the ^1^H-NMR spectrum of **4**, a dominant anomeric signal (**4a**) was accompanied by an additional small one (**4b**). The ^13^C-NMR spectrum of **4a** showed signals corresponding to β-Xyl*p* of protocatechuic acid. Shifts of glycone carbons were compared positively with literature data [51]. Esterification with protocatechuic acid was confirmed by simple acidic and alkaline hydrolysis, as Markham described [51]. Our results indicate that **4a** should be **1-*O*-protocatechuoyl-β-xylose** (Figure 4). As an impurity of **4a**, protocatechuic acid-3-*O*-β-glucoside (**4b**) was deduced.

Besides EA (**16**), *O*-glycosides of ellagic acid were distinguished in Senga Sengana leaf extracts (*m*/*z* 433.0408 and 447.0570). Ellagic acid glycosides fragmented to ions corresponding to EA at *m*/*z* 301 and its decarboxylated monolactone at 275 [LHHDP−44/CO_2_−H]^−^. Analogous pentosides and deoxyhexosides of ellagic acid and methylellagic acid were identified in strawberries from cultivars Alba, Clery, Darselect, Elsanta, Eva, Portola [44], and others.

The next group of compounds characterized by UHPLC comprised phenylethanoids (isomers of 1-*O*-β-[2′-(4″-hydroxyphenyl)ethyl]-6-*O*-(*p*-coumaroyl)glucose) with a pseudomolecular ion [M−H]^−^ at *m*/*z* 445.1507 (calc. 445.1504 for [C_23_H_25_O_9_]^−^) and fragment ions characteristic for *p*-coumaric acid esters: 163 [*p*-coumaric acid−H]^−^, 145 [*p*-coumaroyl−H]^−^ and 119 [*p*-coumaric acid−44/CO_2_−H]^−^. Their UV-VIS spectra exhibited *λ*_max_ at 312 nm, resulting from the HCA moiety. A compound with a pseudomolecular ion 461.1448 (calc. 461.1453 for [C_23_H_25_O_10_]^−^) and *λ*_max_ at 323 nm was tentatively identified as a caffeoyl ester of hydroxyphenylethanol-glycoside. The *cis*/*trans* isomers of eutigoside A and *O*-hydroxyphenylethyl-*O*-caffeoyl-glucoside were reported previously in *F. ananassa* cv. Jonsok [42]. In addition, several ions derived from isomers of *cis*/*trans*-caffeic and *p*-coumaric acid glycosides or esters were also observed [21,43].

### 2.5. Chemical Similarity of the Leaves of F. ananassa cv. Senga Sengana and F. vesca

**Figure 5:**
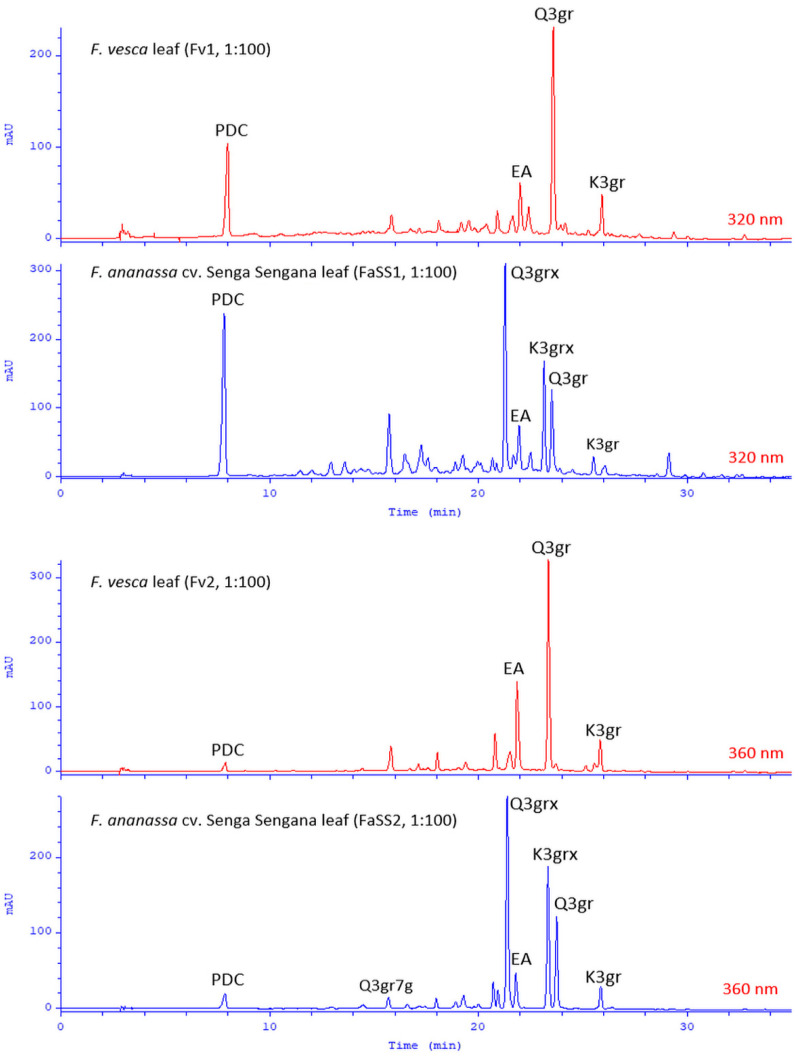
Summary of HPLC-DAD chromatograms attained for water-methanol extracts from *F. vesca* (Fv1, Fv2), and *F. ananassa* cv. Senga Sengana (FaSS1, FaSS2) leaves; λ given in red. Abbreviations: EA, ellagic acid (**16**); K3gr, kaempferol-3-*O*-β-glucuronoside (not isolated); K3grx, kaempferol-3-*O*-[β-xylosyl(1‴→2″)]-β-glucuronoside (**13**); PDC, 2-pyrone-4,6-dicarboxylic acid (**1**); Q3gr, quercetin-3-*O*-β-glucuronoside; Q3grx, quercetin-3-*O*-[β-xylosyl(1‴→2″)]-β-glucuronoside (flagarin; **11**); Q3gr7g, quercetin-3-*O*-β-glucuronoside-7-*O*-β-glucoside (**9**).

The results obtained from qualitative and quantitative examination of *F. ananassa* cv. Senga Sengana leaves (FaSS1, FaSS2, FaSS3) were compared with the corresponding data for *F. vesca* leaves (Fv1 and Fv2). Metabolite profiling showed high similarity between these two species. In woodland strawberry leaves, we observed a comparable PDC content (0.9–1.1% dry matter) and lower levels of ellagitannins and flavonoids. The principal component in both species was agrimoniin (*F. vesca* 1.48–2.70%). Significant differences were observed in flavonol composition, as the quercetin and kaempferol diglycosides typical for *F. ananassa* cv. Senga Sengana were absent in *F. vesca*. Between flavonol derivatives, only 3-*O*-β-glucuronosides were detected, and their content was slightly higher than in the leaves of the garden strawberry (**12**, Q3gr, 0.26–0.53%; K3gr, 0.09–0.12%). We also confirmed the occurrence of other minor components. The chemical composition of Fv1 and Fv2 was largely consistent with the scientific literature data [52]. A summary of HPLC chromatograms of *F. vesca* and *F. ananassa* cv. Senga Sengana leaf extracts is displayed in Figure 5.

### 2.6. Antiglycative and Anti-MGO Effects of Flavonols

Flavonols such as quercetin and kaempferol are known for their properties of trapping reactive carbonyl species (RCS), including methylglyoxal (MGO) and glyoxal (GO) [53]. These dicarbonyls originate from the metabolism of simple sugars such as fructose and glucose (from fructolysis and glycolysis in the liver), as well as from lipid peroxidation, and induce carbonyl stress. RCS, regardless of their sources of origin, react with the amino, guanidine and thiol groups of proteins, modifying their structure (post-translational modification) and physiological functions. They also react with lipoproteins, and purine bases in nucleic acids. These reactions result in harmful advanced glycation end products (AGEs) [54]. MGO, produced in excess in the liver, is subsequently secreted into the systemic circulation. Higher plasma MGO levels have been confirmed in hyperglycemic and dyslipidemic subjects and have been linked to metabolic dysfunction, insulin resistance, type 2 diabetes, diabetic retinopathy and nephropathy, non-alcoholic fatty liver (NAFLD), central obesity, atherosclerosis, gout, and other age-related chronic inflammatory diseases such as cardiovascular disease and disorders of the central nervous system. The high MGO concentration in hepatocytes blocks the allosteric binding of AMP to adenosine monophosphate-activated protein kinase (AMPK). Methylglyoxal can modify three arginines in the gamma subunit of AMPK, resulting in its inactivation. AMPK is an enzyme that controls cellular energy, which functions as an energy sensor for metabolic homeostasis and insulin signaling. When AMPK is inhibited, it thereby favors the anabolic processes, including lipogenesis and insulin resistance, which are related to metabolic syndrome and NAFLD [3]. Therefore, strategies that reduce the MGO level through its uptake (neutralization) are considered appropriate for therapeutic or preventive health care. An example of such an intervention is a randomized, double-blind, placebo-controlled, crossover study with quercetin-3-*O*-β-glucoside (isoquercitrin, 160 mg/day), which observed an 11% reduction in plasma MGO levels in humans [55]. Cardio-metabolic benefits of quercetin in elderly patients with metabolic syndrome were found by Shatylo et al. [56]. The study of Yi et al. [57] summarized quercetin’s therapeutic effects and mechanisms in metabolic diseases.

The two main strawberry quercetin glycosides, Q3gr (**12**, miquelianin) and Q3grx (**11**, flagarin), isolated from Senga Sengana leaves, were evaluated for their antiglycation potential in vitro (BSA-MGO model and MGO trapping assays) against known inhibitors such as quercetin, aminoguanidine, and metformin. Metformin is the primary drug used to treat insulin resistance and diabetes type 2. Other quercetin glycosides commonly found in plant materials, including 3-*O*-β-glucoside (Q3g, isoquercitrin), 4′-*O*-β-glucoside (Q4′g, spireoside), and 3-*O*-β-galactoside (Q3ga, hyperoside), were also used in this experiment. Rutin (quercetin-3-*O*-rutinoside) was examined by us previously [13]. Hyperoside showed the highest antiglycation potential in the assay, and it was comparable to the effect of quercetin (78% vs. 77%) (Table S1). Slightly lower activity (statistically insignificant) was observed for isoquercitrin and miquelianin (**12**) (75% and 72%)—at the aminoguanidine activity level. Next in line were spireoside (64%), metformin (52%), and **11**, quercetin-3-O-[β-xylosyl(1‴→2″)]-β-glucuronoside (45%). 

**Figure 6:**
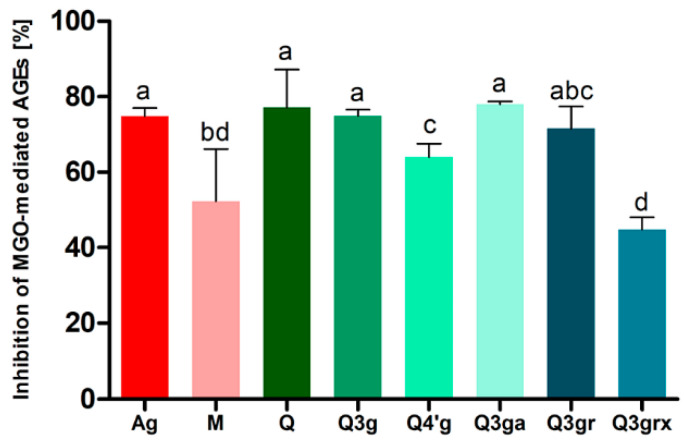
Antiglycation activity after seven days of incubation of BSA with MGO (0.5 mM) and tested compound (1.5 mM), expressed as % inhibition of MGO-mediated-AGE formation. The results are representatives of three experiments performed in triplicate ± SD; Values not sharing a common letter are significantly different at *p* < 0.05 by Tukey’s multiple comparisons test. Abbreviations: Ag, aminoguanidine (reference); M, metformin (reference); Q, quercetin (reference); Q3g, quercetin-3-*O*-β-glucoside (isoquercitrin; minor FaSS extracts constituent); Q4′g, quercetin-4′-*O*-β-glucoside (spireoside; reference); Q3ga, quercetin-3-*O*-β-galactoside (hyperoside; reference); Q3gr, quercetin-3-*O*-β-glucuronoside (miquelianin; **12**); Q3grx, quercetin-3-*O*-[β-xylosyl(1‴→2″)]-β-glucuronoside (flagarin; **11**).

The antiglycation activity of polyphenols is the result of several processes, among which the ability to trap in situ formed RCS, neutralization of reactive oxygen species, as well as reducing and chelating properties, can be considered important. Quercetin and its glycosides are characterized by potent antiradical and reducing effects, and significant antiglycation activity [13]. The type of sugar forming the glycosidic bond and the site of its substitution with the aglycone seem to be essential for this action. Diglycoside **11** (flagarin) was found to be substantially less active compared to the aglycone, but monoglycosides had comparable glycation inhibitory potency (Figure 6), especially those with sugar at the C-3 position (Q3ga = Q ≥ Q3g ≥ Q3gr ≥ Q4′g > Q3grx). It is known that substituting phenolic groups at positions C-5 and C-7 can lose flavonoid MGO trapping potency. Still, position C-4′ (spireoside) only slightly reduces the effect, probably due to the loss of ability to form quinone forms or complexes with metal ions by the catechol group. Interestingly, the 3-*O*-β-glucuronoside of quercetin (**12**, miquelianin) retained high glycation inhibitory activity, which gives hope that the antiglycation capacity of flavonol metabolites can be maintained. Indeed, quercetin-3-*O*-glucuronoside, quercetin-3′-*O*-sulfate, and isorhamnetin-3-*O*-glucuronoside (syn. 3′-*O*-methylquercetin-3-*O*-glucuronoside) have been identified among the phase II metabolites after oral administration of quercetin glycosides [58,59]. All quercetin monoglycosides better protected BSA from modification compared with metformin. However, it should be noted that these compounds may be less available for different human tissues and organs. After oral intake, plasma concentrations of flavonol metabolites are unfortunately much lower compared to metformin. Nevertheless, they may play an important role in inhibiting glycation in the gut. There is known evidence for the endogenous formation of AGEs in the gastrointestinal tract [60].

In another in vitro test, we examined the ability of quercetin glycosides to trap MGO. The quercetin aglycone was used as the reference substance. All compounds formed adducts with methylglyoxal in vitro. However, di-adducts were noted only for quercetin, hyperoside, spireoside, and miquelianin (Q, Q3ga, Q4′g, and Q3gr) (Table 3). The flavonol A-ring arrangement provides the possibility of MGO addition at two positions, and the resulting adducts take the structure of hemiketal or hemiacetal [61]. Therefore, in the reaction products, we observed several forms of isomeric mono- and di-adducts with different retention times but identical masses, higher by 72 Da or 144 Da, respectively, compared to the precursor. Figure S6 shows the corresponding ions for Q3gr (**12**) and Q3grx (**11**) adducts.

Due to the nature of the tannins (their incompatibility with the BSE proteins), this assay was found to be not suitable to measure their suspected antiglycation potential.

## 3. Discussion

Therapeutic uses of wild strawberry leaf preparations include treating gastrointestinal and urinary tract disorders [8]. Possibly, tannins present in leaves exert an astringent effect on the mucosa and have antimicrobial activity [9,62]. On the other hand, flavonoids are responsible for antioxidant, anti-inflammatory, cytoprotective, and diuretic action [63,64,65,66]. The glycation inhibitory and anti-MGO actions of strawberry flavonols may also be crucial for improving liver metabolism, e.g., quercetin and kaempferol glycosides could protect AMPK against post-translational modification and/or activate AMPK [67]. That effect was described in older scientific sources as ‘improving metabolism’ and ‘blood purification’. Thus, polyphenols identified in the Senga Sengana leaf extracts could contribute to its biological effects. For example, Zhang and coworkers’ findings [68] showed that *F. ananassa* leaves significantly alleviate cognitive and memory dysfunction in a diabetic animal model. The garden strawberry leaf components exhibited a strong antioxidant effect, reduced the blood glucose of diabetic rats, and improved their cognitive function by regulating the inflammatory response and inhibiting the caspase cascade. Similarly, *F. ananassa* leaf extract significantly decreased blood glucose, plasma creatinine, urea nitrogen, and renal malondialdehyde in diabetic nephropathy of rats [69]. Kashchenko et al. [70] attributed the hypoglycemic effect of agrimoniin to α-glucosidase inhibition. In experiments by D’Urso et al. [52], an aqueous extract of woodland strawberry leaves possessed direct, endothelium-dependent vasodilatation activity, and its potency was similar to that of an aqueous hawthorn extract. Other researchers reported the inhibitory action of *F. vesca* leaf extract, and the respective ellagitannin-enriched fraction, in *Helicobacter pylori* isolates with differential virulence, suggesting the potential of this plant material for the development of new medicines [71]. Furthermore, Juergenliemk et al. [72] confirmed in vitro that quercetin-3-*O*-glucuronoside (miquelianin) could cross the blood–brain barrier and reach the CNS, which determines its antidepressant effects demonstrated in animal model studies.

Therefore, based on our results and the cited scientific data, we conclude that the compounds identified in the leaves of *F. ananassa* and *F. vesca* are bioactive components. Their occurrence explains the biological effects observed in the quoted experiments, which may partially be due to the ability of flavonols to trap MGO and inhibit the harmful glycation of biomolecules. However, the antiglycation properties of flavonol glycosides demonstrated in this study are only preliminary and fraught with the inherent limitations of an in vitro model. Their translation to a therapeutic or prophylactic effect in humans requires further studies, including clinical trials.

In recent years, significant emphasis has been placed on the zero-waste way of life. As a result, different branches of industry, farming, and cultivation are forced to be more productive in a green manner. Some of the prominent examples are mainly connected with cereals or fruit processing, including valorization of by-products [73,74,75,76,77] and technological solutions [78,79]; however, more holistic approaches are found, too [80,81]. Our findings show that waste materials resulting from strawberry production should attract the attention of pharmaceutical companies.

## 4. Materials and Methods

### 4.1. Chemicals

LC-MS grade solvents were from Merck (Darmstadt, Germany). Analytical grade diisopropyl ether was purchased from Sigma-Aldrich (St. Louis, MO, USA). Other analytical grade solvents were from POCh (Lublin, Poland). Deuterated solvents were from Armar AG (Döttingen, Switzerland). Methylglyoxal (MGO, 40% in water), bovine serum albumin (BSA, ≥98%), and sodium azide (99%) were purchased from Sigma-Aldrich/Merck; MGO-assay salts (reagent grade) were from Chempur (Piekary Śląskie, Poland).

### 4.2. Standards

Kaempferol, quercetin, myricetin, taxifolin, catechin, epicatechin, astragalin (K3g, kaempferol-3-*O*-β-glucoside), kaempferol-3-*O*-β-glucuronoside (K3gr), nicotiflorin (K3grh, kaempferol-3-*O*-rutinoside), tiliroside (T, kaempferol-3-*O*-β-(6″-*O*-*p*-coumaroyl) glucoside), isoquercitrin (Q3g, quercetin-3-*O*-β-glucoside), quercetin-4′-*O*-β-glucoside (Q4′g, spireoside or spiraeoside), hyperoside (Q3ga, quercetin-3-*O*-β-galactoside), gallic acid (GA), protocatechuic acid (PA), caffeic acid (CA), and *p*-coumaric acid (pCuA) with declared purity 99% were from Extrasynthese (Genay, France). Ellagic acid (EA, ≥95%) was purchased from Koch-Light Laboratories (Colnbrook, UK), while rutin (Q3grh, quercetin-3-*O*-rutinoside), citric acid, quinic acid, and sugar standards were from Merck. Agrimoniin (A) with approximately 97% purity (HPLC-DAD at λ 254 nm) was previously isolated from *Potentilla anserina* L. [11]. Procyanidins B3 and C2 with approximately 95% purity (HPLC-DAD at λ 254 nm) were isolated from *Potentilla erecta* (L.) Raeusch. [33]. Isolated compounds analyzed in the antiglycation assay (**11**, **12**) were of at least 95% purity (HPLC-DAD at λ 254 nm, UHPLC-MS).

Stock standard solutions of polyphenols at 1 mg/mL concentration were prepared in methanol, except the ellagic acid (prepared in dimethyl sulfoxide). Working standard solutions (20–800 μg/mL) were prepared by dilution with 50% water-methanol (*v*/*v*), then filtered through Durapore 0.22 μm syringe filters (Millipore; Burlington, MA, USA), and stored in the freezer (−20°C).

Metformin hydrochloride (M; Pharmaceutical Secondary Standard) and aminoguanidine hydrochloride (Ag; ≥98%) were purchased from Sigma-Aldrich/Merck. The stock solutions of compounds for in vitro assays (3 mM) were prepared by dissolving reference compound in 5 mL of a suitable solvent and filtered through hydrophilic Millex as above.

### 4.3. Plant Material

Leaves with petioles of garden strawberry (*Fragaria × ananassa* Duch. cv. Senga Sengana) were collected from the local plantation in Pasikurowice (district Długołęka, Poland) in July of two consecutive cultivation years (FaSS1, FaSS2; 5 kg each) and from the experimental farm COBORU (Research Centre for Cultivar Testing, Masłowice, Poland) in July of 2020 (FaSS3; 1 kg). The leaves were collected as agricultural waste after fruit harvesting. Before extraction, leaves were air-dried at ambient temperature (22 ± 2 °C), protected from direct sunlight, and crushed. Plant voucher specimens designated as FaSS1, FaSS2, and FaSS3, respectively, are retained in the Herbarium of our Department (Faculty of Pharmacy, Wroclaw Medical University).

Dry leaves of woodland strawberry (*Fragaria vesca* L.) harvested in July from the Botanical Garden of the University of Wrocław (Poland) were applied as reference material Fv1. A commercial woodland strawberry leaf Fv2 (Flos, Mokrsko, Poland) was used in the comparative analysis.

### 4.4. Sample Preparation for LC

For the LC analysis, 50% water-methanol extracts (DER 1:100; *m*/*v*) were prepared from 0.5 g samples of powdered strawberry leaves and water-methanol mixture (1+1, *v*/*v*) in an ultrasonic bath Sonorex Digital 10P (Bandelin, Berlin, Germany). Extraction was performed at an ambient temperature for 15 min [11]. The resulting extracts were transferred to volumetric flasks, brought up to 50 mL with an extraction solvent, and next filtered through Whatman filter papers Grade 1 (Little Chalfont, UK). Fractions, sub-fractions, and isolated compounds were dissolved with water-methanol (1+1, *v*/*v*). Samples of all analyzed extracts and solutions were subsequently filtered by syringe filters (Durapore 0.22 μm).

### 4.5. Isolation of F. ananassa cv. Senga Sengana Leaf Constituents

Dry strawberry leaves of cultivar Senga Sengana (FaSS1, 500 g) were processed chromatographically to yield the following constituents: **1** (235 mg), **2** (386 mg), **3** (420 mg), **4** (4a, 65 mg), **5** (464 mg), **6** (195 mg), **7** (2533 mg), **8** (11 mg), **9** (27 mg), **10** (5817 mg), **11** (330 mg), **12** (216 mg), **13** (48 mg), **14** (25 mg), **15** (17 mg), and **16** (245 mg). Detailed data on the separation process of these components are shown in Figure S1. Retention parameters of isolated compounds together with tentative UHPLC-qTOF-MS/MS identification of non-isolated compounds from FaSS are given in Table 1.

### 4.6. Identification and Quantification

Structures of compounds 1, 3–5, 7–15 were characterized by spectroscopic evidence (UV-VIS, ESI-MS, 1D- and 2D-NMR) and compared with literature. Compounds **2**, **6**, and **16** were compared chromatographically (R_f_, t_R_), UV-VIS (*λ*_max_), and ESI-MS (*m*/*z*) with authentic standards. Compounds **1**–**3** and **5**–**16** were free from other phenolic impurities (purity ≥ 95%, HPLC-DAD; Section 4.6.2). Compound **4a** was accompanied by a dopant of a close derivative **4b**. Details of NMR and UV-VIS are available in Supplementary Materials, while HRMS data are in Table 1, along with retention data.

#### 4.6.1. Structure Elucidation Equipment

One-dimensional- and two-dimensional-NMR experiments were performed on Bruker Avance 300 MHz and 500 MHz spectrometers (Bruker BioSpin, Rheinstetten, Germany) using the residual solvent peaks as internal standards. NMR tests for glycosides were conducted in DMSO-*d6*, ellagitannins in acetone-d6 with D_2_O (1+1, *v*/*v*), and acids and esters in CD_3_OD. NMR data were analyzed by MestReNova 12 software (Mestrelab Research, Santiago de Compostela, Spain). Direct MS spectra (in negative mode) were recorded in water-methanol (1+1, *v*/*v*) on the ESI-qTOF Compact mass spectrometer (Bruker Daltonics, Bremen, Germany). LC-MS-derived spectra were recorded in an appropriate eluent (water-methanol acidified with formic acid) in negative mode. MS data were managed by Data Analysis 4.2 software (Bruker Daltonics). UV-VIS spectra of isolated compounds were measured in water-methanol (1+1, *v*/*v*; 0.01–0.03 mM) on a Cecil CE 3021 spectrometer (Cecil Instruments, Cambridge, UK).

#### 4.6.2. Chromatography 

TLC was performed on silica gel 60 plates with a fluorescent indicator (Si60 F254, 0.25 mm, 10 × 20 cm; Merck) at ambient temperature. Tested solutions (5–35 μL) were applied as 3–5 mm bands and chromatographed in mobile phases consisting of diisopropyl ether, acetone, formic acid, and water (ellagitannins-**E**, 4+4+1+1; flavonoids-**F**, 5+3+1+1; phenolic aids-**P**, 5+2+2+1; *v*/*v*/*v*/*v*) [82]. Simple sugars (monosaccharides), liberated during acid hydrolysis, were identified in the mobile phase **S**-chloroform-methanol-glacial acetic acid-water (8+5+0.2+0.8; *v*/*v*/*v*/*v*), whereas flavonoid aglycones were identified in the mobile phase **X** (chloroform-acetone-water, 5+7+8; *v*/*v*/*v*) and **Z** (chloroform-acetone-formic acid, 8+1+1; *v*/*v*/*v*). Flavonoids were visualized at 254 nm and 366 nm, without and with AlCl_3_ (2% in methanol) or NP (1% in methanol) spraying. Hydrolyzable tannins and phenolic acids were visualized in VIS using FeCl_3_ (1% in methanol), whereas monosaccharides with a thymol reagent (0.5 g of thymol in a mixture of 5 mL of concentrated H_2_SO_4_ with 95 mL of methanol; heated at 120 °C).

The validated HPLC-DAD method described previously was used to examine the components of strawberry leaf extracts [33]. Separation was achieved on a Hypersil Gold C18 column (250 × 4.6 mm, ∅ 5 μm) with a C18 precolumn (10 × 4.6 mm, ∅ 5 μm) (Thermo Fisher Scientific, Waltham, MA, USA). The linearity of the HPLC-DAD method and quantification of selected polyphenols and PDC was performed based on regression equations determined for the isolated compounds from peak areas and corresponding concentrations.

The Ultimate 3000 system (Thermo Fisher Scientific) with a Kinetex C18 column (150 × 2.1 mm, ∅ 2.6 μm for extracts; 100 × 2.1 mm, ∅ 2.6 μm for MGO derivatives; Phenomenex, Torrance, CA, USA) coupled to the ESI-qTOF Compact mass spectrometer (Bruker Daltonics) was used for qualitative UHPLC-qTOF-MS/MS. Equipment, analysis parameters, and gradient were as reported previously [82](for analysis of extracts), [13](for analysis of MGO adducts).

#### 4.6.3. Acid Hydrolysis

Solutions of isolated hydrolyzable tannins (∼50 mg in 5 mL of 5% H_2_SO_4_) were heated (90 °C, reflux, eight hours) according to Tanaka et al. [83]. The hydrolysis products were purified on the octadecyl columns (Isolute C18, 10 g, Biotage, Uppsala, Sweden), then concentrated under reduced pressure and diluted in methanol (5 mL). Phenolic acids and depsides released by hydrolytic degradation were analyzed by TLC (mobile phase **P**).

Solutions of other polyphenols isolated in small amounts (1 mg in 1 mL 5% H_2_SO_4_) were heated at 90 °C in sealed vials for four hours. The hydrolysis products were separated by adding ethyl acetate into water residues [51]. Water layers, concentrated and re-dissolved in 1 mL of methanol, were examined for sugars (TLC, mobile phase **S**). Organic layers, concentrated and re-dissolved in 1 mL of methanol, were investigated for aglycones (TLC, mobile phases **X** and **Z**).

#### 4.6.4. Alkaline Hydrolysis

To initially differentiate the 3-*O*- and 7-*O*- position of glycosylation, a small quantity of isolated polyphenol (1 mg) was subjected to alkaline hydrolysis in 1 mL of 0.5% (~0.09 M) aqueous KOH in a boiling water bath for three hours [84].

### 4.7. Inhibition of Glycation and MGO Trapping In Vitro

#### 4.7.1. Antiglycation Assay in BSA-MGO Model

The formation of advanced glycation end-products (AGEs) was measured following a slightly modified method proposed by Liu et al. [85]. In brief, 21.2 μM bovine serum albumin was incubated with methylglyoxal (MGO) at 0.5 mM in 100 mM PBS at pH 7.4 with 0.02% sodium azide (for prevention of microbial growth). The compounds investigated for inhibition of non-enzymatic glycation were added at a final concentration of 1.5 mM. Then, the reaction solution was incubated at 37 °C and shaken at 50 revolutions per minute for seven days in closed vials away from light. Measurement of the fluorescent intensity of total AGEs after incubation was carried out using a Synergy HTX Multi-Mode Microplate Reader (BioTek Instruments Inc., Winooski, VT, USA) at a wavelength of 360 nm for excitation and 460 nm for emission. Data acquisition was obtained with the Gen5 Software (BioTek Instruments Inc., Winooski, VT, USA). The measurements from three experiments were all performed in triplicate, and the percentage inhibition of AGE formation was calculated using the following equation:

Inhibition of AGE formation = [1 − (Fls/Fl_0_)] × 100 [%], (1)

where Fl_0_ is the mean fluorescence intensity of the blank sample and Fls is the mean fluorescence intensity of the sample.

#### 4.7.2. MGO Trapping Assay

Methylglyoxal trapping activity was investigated according to a slightly modified version of the Sang et al. [86] method, as previously described [13]. Briefly, 0.6 mM methylglyoxal was incubated for one hour with 0.2 mM of each compound in 100 mM PBS at pH 7.4 and 37 °C to equate to physiological conditions and shaken at 50 revolutions per minute. The incubation reaction was stopped by adding 2.5 μL of acetic acid and placing the collected samples in an ice-cold water bath. Next, the samples were filtered through hydrophilic syringe filters (Durapore 0.22 μm) and analyzed using UHPLC-qTOF-MS/MS to investigate their ability to form adducts with methylglyoxal. The trapping agent solutions were freshly prepared before each series of experiments was begun, and the pH of the sodium phosphate buffer was determined immediately before use.

### 4.8. Statistical Analysis 

All data are presented as means ± standard deviation (SD). Data were analyzed using the Shapiro–Wilk test to assess normality of distribution, followed by one-way analysis of variance (ANOVA) with Tukey’s multiple comparison test using the GraphPad Prism 6 software; P values equal to or less than 0.05 were considered significant.

## 5. Conclusions

To summarize, a detailed chemical analysis of *F. ananassa* cv. Senga Sengana leaves revealed the presence of a broad spectrum of polyphenolic metabolites. Their major components were ellagitannins. Galloyl esters of quinic acid and glucuronosides of flavonols constituted the second group. Proanthocyanidins, flavan-3-ols, and phenolic acid derivatives were minor components. Several new polyphenols including kaempferol and quercetin xylosyl-glucuronosides were also identified. 

The occurrence of polyphenols together with 2-pyrone-4,6-dicarboxylic acid at relatively high levels further provides the opportunity to utilize this resource in biotechnological processes to obtain PDC. Moreover, strawberry flavonols have demonstrated the capacity to uptake methylglyoxal and inhibit protein glycation in vitro. Due to that, the leaves of Senga Sengana may become a new raw plant material with therapeutic, pro-health, or cosmetic phytoconstituents.

## Figures and Tables

**Table 1:** Identification of *F. ananassa* cv. Senga Sengana leaf components by UPLC-qTOF-MS/MS.

*t_R_* [min]	UV *λ*_max_ [nm]	MS^1^[M−H]^−^ [*m*/*z*] Measured	MS^1^[M−H]^−^ [*m*/*z*] Calculated	MS^2^[M−H]^−^ [*m*/*z*] Measured	Identification	Compound, Reference
Carboxylic acids						
1.76	315	182.9934, 366.9940 [2M−H]^−^	182.9935	139 [M−44/CO_2_−H]^−^, 111 [M−44/CO_2_−28/CO−H]^−^	2-pyrone-4,6-dicarboxylic acid, PDC	(**1**), isol, [25]
1.32	-	191.0198	191.0197	111	citric acid (isomers)	std
1.45						
1.15	-	191.0563	191.0561	173 [M−18/H_2_O−H]^−^, 155 [M−36/2H_2_O−H]^−^, 111 [M−44/CO_2_−36/2H_2_O−H]^−^	quinic acid	(**2**), std
Hydrolyzable tannins						
7.24	275	331.0669	331.0671	169 [GA−H]^−^, 125 [GA−44/CO_2_−H]^−^	galloyl-glucose, e.g., *1-O-galloyl-α-glucose*	[27,28]
1.21	273	343.0669, 687.1407 [2M−H]^−^	343.0671	191 [QA−H]^−^, 169 [GA−H]^−^, 125 [GA−44/CO_2_−H]^−^	*O*-galloylquinic acid (isomer 1)	[27,28]
1.52					3-*O*-galloylquinic acid (isomer 2)	(**3**), isol, [14]
3.02				191 [QA−H]^−^, 169 [GA−H]^−^, 125 [GA−44/CO_2_−H]^−^	5-*O*-galloylquinic acid, *theogallin* (isomer 3)	(**5**), isol, [14]
3.74				191 [QA−H]^−^, 173 [QA−18/H_2_O−H]^−^, 169 [GA−H]^−^, 125 [GA−44/CO_2_−H]^−^	*O*-galloylquinic acid (isomer 4)	[14]
11.75	275	483.0770	483.0780	169 [GA−H]^−^, 125 [GA−44/CO_2_−H]^−^	digalloyl-glucose	[27,28]
11.19	273	495.0778	495.0780	343 [M−152/gall−H]^−^, 191 [QA−H]^−^, 169 [GA−H]^−^, 125 [GA−44/CO_2_−H]^−^	3,5-*O*-digalloylquinic acid (or isomer)	[14]
9.03	242, 275	633.0724	633.0733	481 [M−152/gall−H]^−^, 463 [M−152/gall−18/H_2_O−H]^−^, 301 [EA−H]^−^, 275 [LHHDP−44/CO_2_−H]^−^, 169 [GA−H]^−^, 125 [GA−44/CO_2_−H]^−^	galloyl-HHDP-glucose (isomers), e.g., *sanguiin H-4*	[27,28]
10.79						
13.54	275	635.0894	635.0890	465 [M−152/gall−18/H_2_O−H]^−^, 313 [M−304/2gall−18/H_2_O−H]^−^, 169 [GA−H]^−^, 125 [GA−44/CO_2_−H]^−^	trigalloylglucose	[27,28]
2.88	243	783.0676	783.0686	481 [M−302/HHDP−H]^−^, 301 [EA−H]^−^, 275 [LHHDP−44/CO_2_−H]^−^	pedunculagin (isomers), e.g., *α*/*β anomers*	(**7a**) and (**7b**), isol, [26,29,30]
5.36						
8.68						
10.20	273	785.0826	785.0843	633 [M−152/gall−H]^−^, 481 [M−304/2gall−H]^−^, 301 [EA−H]^−^, 275 [LHHDP−44/CO_2_−H]^−^, 169 [GA−H]^−^, 125 [GA−44/CO_2_−H]^−^	digalloyl-HHDP glucose	[28]
11.81	345	933.0656 466.0270 [M−2H]^−2^	933.0640, 466.0283 [M−2H]^−^	631[M−302/HHDP−H]^−^, 481 [M−452/VTL−H]^−^, 451 [VTL−H]^−^, 301 [EA−H]^−^, 275 [LHHDP−44/CO_2_−H]^−^, [GA−H]^−^, 125 [GA−44/CO_2_−H]^−^	ellagitannin-934 (isomers), e.g., *α*/*β anomers of praecoxin D*	[30,31]
12.98						
13.17	275	935.0808, 467.0354 [M−2H]^−2^	935.0796, 467.0362 [M−2H]^−^	783 [M−152/gall−H]^−^, 633 [M−302/HHDP−H]^−^, 613 [M−304/2gall−18/H_2_O−H]^−^, 481 [M−302/HHDP−152/gall−H]^−^, 463 [M−302/HHDP−152/gall−18/H_2_O−H]^−^, 313 [M−604/2HHDP−18/H_2_O−H]^−^, 301 [EA−H]^−^, 275 [LHHDP−44/CO_2−_H]^−^, 169 [GA−H]^−^, 125 [GA−44/CO_2_−H]^−^	potentillin	[28,30]
14.47	243	1235.0744, 617.0299 [M−2H]^−2^	1235.0702, 617.0315 [M−2H]^−2^	933 [M−302/HHDP or DHDG−H]^−^, 935 [M−300−H]^−^ (potentillin), 613 [M−302/HHDP−302/DHDG−18/H_2_O−H]^−^, 783 [M−452/VTL−H]^−^, 481 [M−452/VTL−302/HHDP or DHDG−H]^−^, 469 [val−H]^−^, 463 [M−302/HHDP or DHDG−470/val−H]^−^, 451 [VTL−H]^−^, 319 [LDHDG−H]^−^, 301 [EA−H]^−^, 275 [LHHDP−44/CO_2_−H]^−^, [GA−H]^−^, 125 [GA−44/CO_2_−H]^−^	ellagitannin-1236, e.g., *davuriciin D2* / *fragariin A lactone without a pedunculagin fragment*	[28,30,32]
12.31	245, 276	783.0672 [M−2H]^−2^	1567.1446, 783.0686 [M−2H]^−2^	1265 [M−302/HHDP−H]^−^ (laevigatin E), 1103 [M−302/HHDP−162/glc−H]^−^ (agrimonic acid A/B), 1059 [M−302/HHDP−162/glc−44/CO_2_−H]^−^, 935 [M−632−H]^−^ (potentillin), 783 [M−784/pedunculagin−H]^−^ (pedunculagin or laevigatin A lctone), 633 [M−934−H]^−^, 613 [M−936−18/H_2_O−H]^−^, 481 [M−1086 /agrimonic acid lactone−H]^−^, 463 [M−1086/agrimonic acid lactone−18/H_2_O−H]^−^, 319 [LDHDG−H]^−^, 301 [EA−H]^−^, 275 [LHHDP−44/CO_2_−H]^−^, 125 [GA−44/CO_2_−H]^−^	laevigatin B/C/F (isomers)	[33,34,35]
13.17						
13.93						
14.33	245	934.0722 [M−2H]^−2^	1869.1508, 934.0718 [M−2H]^−2^	1567 [M−302/HHDP−H]^−^ (laevigatin B/C/F), 1085 [M−784/ pedunculagin−H]^−^, 935 [M−934−H]^−^ (potentillin), 897 [M−936/ potentillin−36/2H_2_O−H]^−^, 783 [M−1086/agrimonic acid A/B lactone−H]^−^ (pedunculagin) or [M−784/ pedunculagin−302/HHDP−H]^−^ (laevigatin A lactone), 745 [M−936/potentillin−152/gall− 36/2H_2_O)−H]^−^, 633 [M−1236−H]^−^, 613 [M−1086/agrimonic acid lactone A/B−152/gall−18/H_2_O−H]^−^, 481 [M−1086/agrimonic acid A/B lactone−302/HHDP−H]^−^, 463 [M−1086(agrimonic acid A/B lactone)−302(HHDP)−18(H_2_O)−H]^−^, 319 [LDHDG−H]^−^, 301 [EA−H]^−^, 275 [LHHDP−44/CO_2_−H]^−^, 125 [GA−44/CO_2_−H]^−^	agrimoniin	(**10**), isol, [11,29,33]
16.99	243	1009.0686 [M−2H]^−2^	2019.1461, 1009.0694 [M−2H]^−2^	1235 [M−784/pedunculagin−H]^−^, 933 [M−784/pedunculagin-302/DHDG−H]^−^ (praecoxin D), 897 [M−1104/agrimonic acid B−18/H_2_O−H]^−^, 783 [M−1236−H]^−^ (pedunculagin or laevigatin A lactone), 769 [M−1252−H]^−^, 633 [M−934−452/VTL−H]^−^ or [M−1084−302/HHDP−H]^−^, 613 [M−1104/agrimonic acid B-302/ HHDP−H]^−^, 481 [M−1086/agrimonic acid lactone−452/VTL−H]^−^, 451 [VTL−H]^−^, 319 [LDHDG−H]^−^, 301 [EA−H]^−^, 275 [LHHDP−44/CO_2_−H]^−^, 169 [GA−H]^−^, 125 [GA−H]^−^	ellagitannin-2020, e.g., *davuriciin D2*/*fragariin A lactone*	[27,28,36]
14.23	244	1018.0749 [M−2H]^−2^	2037.1567, 1018.0747 [M−2H]^−2^	1567 [M−470/val−H]^−^ (laevigatin B/C/F), 933 [M−1104/agrimonic acid B−H]^−^, 783 [M−1236−18/H_2_O−H]^−^ (pedunculagin or laevigatin A lactone), 745 [M−1104/ agrimonic acid B−152/gall−36/2H_2_O−H]^−^, 633 [M−934−452/VTL−18/H_2_O−H]^−^ or [M−1084−302/HHDP−18/H_2_O−H]^−^, 613 [M−1236−152/gall−36/2H_2_O−H]^−^, 481 [M−1086−470/val−H]^−^, 463 [M−1086−470/val−18/H_2_O−H]^−^, 451 [VTL−H]^−^, 319 [LDHDG−H]^−^, 301 [EA−H]^−^, 275 [LHHDP−44/CO_2_−H]^−^, 169 [GA−H]^−^, 125 [GA−44/CO_2_−H]^−^	davuriciin D_2_/fragariin A	[27,28,32,36,37]
Proanthocyanidins (condensed tannins) and flavan−3−ols						
10.86	242, 278	289.0719	289.0712	245 [C_13_H_10_O_5_−H]^−^, 205 [C_11_H_10_O_4_−H]^−^, 203 [C_11_H_8_O_4_−H]^−^, 151 [C_8_H_8_O_3_−H]^−^,137 [C_7_H_6_O_3_−H]^−^, 125 [C_6_H_6_O_3_−H]^−^, 123 [C_7_H_8_O_2_−H]^−^, 121 [C_7_H_6_O_2_−H]^−^, 109 [C_6_H_6_O_2_−H]^−^	catechin	(**6**), isol, std, [26]
15.55	242, 278	451.1237	451.1246	289 [M−162/hex−H]^−^	(epi)catechin-*O*-hexoside	-
9.78	242, 278	577.1346	577.1352	451 [M−126/C_6_H_6_O_3_−H]^−^, 425 [M−152/C_8_H_8_O_3_−H]^−^, 407 [M−152/ C_8_H_8_O_3_−18/H_2_O−H]^−^, 289 [M−288/C_15_H_12_O_6_−H]^−^, 287 [M−290/ C_15_H_14_O_6_−H]^−^, 245 [C_13_H_10_O_5_−H]^−^, 137 [C_7_H_6_O_3_−H]^−^, 125 [C_6_H_6_O_3_−H]^−^	catechin dimer, e.g., *procyanidin B3*	std, [26,33]
10.13				425 [M−152/C_8_H_8_O_3_−H]^−^, 407 [M−152/C_8_H_8_O_3_−18/H_2_O−H]^−^, 289 [M−288/C_15_H_12_O_6_−H]^−^, 287 [M−290/C_15_H_14_O_6_−H]^−^, 137 [C_7_H_6_O_3_−H]^−^, 125 [C_6_H_6_O_3_−H]^−^	(epi)catechin dimer (isomers)	[27,28,30,36]
10.23						
11.77						
13.22						
10.13	242, 278	865.1981	865.1985	739 [M−126/C_6_H_6_O_3_−H]^−^, 713 [M−152/C_8_H_8_O_3_−H]^−^, 695 [M−152/ C_8_H_8_O_3_−18/H_2_O−H]^−^, 577 [M−288/C_15_H_12_O_6_−H]^−^, 575 [M−290/ C_15_H_14_O_6_−H]^−^, 451 [M−288/C_15_H_12_O_6_−126/ C_6_H_6_O_3_−H]^−^, 425 [M−288/C_15_H_12_O_6_−152_/_C_8_H_8_O_3_−H]^−^, 407 [M−288/C_15_H_12_O_6−_152/C_8_H_8_O_3_−18/H_2_O−H]^−^, 289 [M−576/C_30_H_24_O_12_−H]^−^, 287 [M−578/C_30_H_26_O_12_−H]^−^, 245 [C_13_H_10_O_5_−H]^−^, 137 [C_7_H_6_O_3_−H]^−^, 125 [C_6_H_6_O_3_−H]^−^	catechin trimer, e.g., *procyanidin C3*	std, [33]
10.66					(epi)catechin trimer (isomers)	[27,28,30,33,36]
11.49						
10.92	242, 278	1153.2610	1153.2619	1027 [M−126/C_6_H_6_O_3_−H]^−^ 1001 [M−152/C_8_H_8_O_3_−H]^−^, 983 [M−152/ C_8_H_8_O_3_−18/H_2_O−H]^−^, 865 [M−288/C_15_H_12_O_6_−H]^−^, 863 [M−290/ C_15_H_14_O_6_−H]^−^, 695 [M−288/C_15_H_12_O_6_−152/ C_8_H_8_O_3_−18/H_2_O−H]^−^, 577 [M−576/C_30_H_24_O_12_−H]^−^, 575 [M−578/C_30_H_26_O_12_−H]^−^, 451 [M−576/C_30_H_24_O_12_−126/C_6_H_6_O_3_−H]^−^, 425 [M−576/C_30_H_24_O_12_− 152/C_8_H_8_O_3_−H]^−^, 407 [M−576/ C_30_H_24_O_12_−152/C_8_H_8_O_3_−18/H_2_O−H]^−^, 289 [M−864/C_45_H_36_O_18_−H]^−^, 287 [M−866/C_45_H_38_O_18_−H]^−^, 137 [C_7_H_6_O_3_−H]^−^, 125 [C_6_H_6_O_3_−H]^−^	(epi)catechin tetramer	
Flavonoids						
17.08	290	435.0930	435.0933	303 [M−132/pent−H]^−^, 285 [M−162/glc−18/H_2_O−H]^−^, 179, 151	taxifolin-*O*-pentoside (isomers), e.g., *taxifolin-3-O-α-arabinoside*	[26]
21.03						
19.23	265, 345	447.0929	447.0933	284/285 [M−162/glc−H]^−^, 229	kaempferol-3-*O-*β-glucoside (astragalin)	std, [21]
19.19	265, 345	461.0724	461.0726	285 [M−176/glcA−H]^−^, 255, 229, 151	kaempferol-3-*O-*β-glucuronoside	std, [28,36]
17.91	255, 353	463.0878	463.0882	301 [M−162/glc−H]^−^, 179, 151	quercetin-3-*O*-β-glucoside (isoquercitrin)	std
10.61	226, 289	465.1035	465.1039	303 [M−162/glc−H]^−^, 285 [M−162/glc−18/H_2_O−H]^−^, 151	taxifolin-*O*-hexoside (isomer 1)	[21]
14.23					(2*R*,3*R*)-taxifolin 3-*O*-β-glucoside (isomer 2)	(**8**), isol, [21]
15.18					taxifolin-*O*-hexoside (isomer 3)	[21]
15.77					taxifolin-*O*-hexoside (isomer 4)	
17.74	255, 353	477.0672	477.0675	301 [M−176/glcA−H]^−^, 179, 151	quercetin-3-*O*-β-glucuronoside (miquelianin)	(**12**), isol, [21,28,36]
19.18	239, 255, 353	491.0806	491.0831	315 [M−176/glcA−H]^−^, 300 [M−176−15/Me^•^−H]^•−^, 137	isorhamnetin-3-*O-*β-glucuronoside	(**14**), isol, [28,38]
17.94	265, 347	593.1144	593.1148	285 [M−308−H]^−^, 229, 151	kaempferol-3-*O*-[β-xylosyl(1‴→2″)]-β-glucuronoside	(**13**), isol, [27,28]
22.07	264, 312, 347	593.1299	593.1300	447 [M−146/*p*-coumaroyl−H]^−^, 285 [M−146/*p*-coumaroyl−162/glc−H]^−^, 145 [*p*-coumaroyl−H]^−^, 119 [*p*CouA−44/CO_2_−H]^−^	tiliroside (*cis*/*trans* isomers)	(**15a**) and (**15b**), isol, [39,40,41,42]
22.25						
16.59	256, 353	609.1108	609.1097	301 [M−308−H]^−^, 179, 151	quercetin-3-*O*-[β-xylosyl(1‴→2″)]-β-glucuronoside, (flagarin)	(**11**), isol, [21,27,28]
13.01	256, 352	639.1200	639.1203	463 [M−176/glcA−H]^−^, 301 [M−176−162/glc−H]^−^, 179, 151	quercetin-3-*O*-β-glucuronoside-7-*O*-β-glucoside	(**9**), isol, [28]
15.77	255, 351			301 [M−338−H]^−^, 179, 151	quercetin-3-*O*-(β-glucosyl)-β-glucuronoside	[28]
9.34	227	285.0614	285.0616	152/153 [M−132/xyl−H]^−^, 108/109 [PA−44/CO_2_−H]^−^	1-*O*-protocatechuoyl-β-xylose	(**4a**), isol
Phenolic acids’ derivatives						
18.18	253, 367	300.9987	300.9990	301 [EA−H]^−^, 275 [LHHDP−44/CO_2_−H]^−^	ellagic acid	(**16**), isol, std
6.14	282	315.0718	315.0722	153 [M−162/glc−H]^−^, 109 [PA−44/CO_2_−H]^−^	protocatechuic acid 3-*O-*β-glucoside	(**4b**), isol
11.35	320	325.0927	325.0929	163 [M−162/hex−H]^−^, 145 [*p*-coumaroyl−H]^−^, 119 [*p*CuA−44/CO_2_−H]^−^	*p*CuA hexoside or ester (isomers), e.g., *p-coumaric acid 4-O-β-glucoside, 1-O-p-coumaroyl-β-glucose*	[21,28,36,43]
11.81						
12.21						
13.13						
14.54						
19.20						
8.14	322	341.0878	341.0878	179 [M−162/hex−H]^−^, 161 [caffeoyl−H]^−^, 135 [CA−44/CO_2_−H]^−^	CA hexoside or ester (isomers), e.g., *1-O-caffeoyl-β-glucose*	[43]
9.03						
10.64						
11.04						
11.42						
12.62						
7.57	322	355.0665	355.0671	179 [M−176/glcA−H]^−^, 161 [caffeoyl−H]^−^, 135 [CA−44/CO_2_−H]^−^	CA glucuronoside or ester (isomers)	
8.94						
9.41						
10.30						
11.09						
12.65						
17.48	253, 361	433.0408	433.0412	301 [EA−132/pent−H]^−^, 275 [LHHDP−44/CO_2_−H]^−^	ellagic acid *O-*pentoside	[28,36]
19.50	246, 312	445.1507	445.1504	353, 236, 205, 163 [*p*CuA−H]^−^, 145 [*p*-coumaroyl−H]^−^, 119 [*p*CuA−44/CO_2_−H]^−^	eutigoside A (isomers)	[28]
19.78						
18.01	245, 377	447.0570	447.0569	301 [M−146/dhex−H]^−^, 275 [LHHDP−44/CO_2_−H]^−^	ellagic acid *O-*deoxyhexoside	[28,36,44]
18.12	246, 285, 323	461.1448	461.1453	179 [M−162/hex−H]^−^, 161 [M−162/hex−138/hydroxyphenylethanol−H]^−^, 135 [CA−44/CO_2_−H]^−^	*O*-hydroxyphenylethyl-*O*-caffeoyl-glucoside	[42]

CA, caffeic acid; dhex, deoxyhexose; DHDG, dehydrodigalloyl; EA, ellagic acid; GA, gallic acid; gall, galloyl; glc, glucose; glcA, glucuronic acid; HHDP, hexahydroxydiphenoyl; hex, hexoside; isol, isolated compound, with (number reference to the text); LDHDG, dehydrodigallic acid monolactone; LHHDP, hexahydroxydiphenic acid monolactone; Me^•^, methyl (radical); QA, quinic acid; PA, protocatechuic acid; *p*CuA, *p*-coumaric acid; pent, pentose; std, standard compound; val, valoneoyl; VTL, valoneic acid trilactone; xyl, xylose; Main [M−H]^−^ signals are underlined.

**Table 2:** Mean content of main compounds (mg/g dry matter) identified in leaves of *F. ananassa* cv. Senga Sengana from three different harvest years.

Compound	Abbreviation	FaSS1		FaSS2		FaSS3	
		Mean	SD	Mean	SD	Mean	SD
2-pyrone-4,6-dicarboxylic acid (**1**)	PDC	13.73	*0.31*	10.37	*0.28*	17.45	*0.45*
5-*O*-galloylquinic acid (**5**)	5gQA	5.21	*0.27*	4.10	*0.16*	6.43	*0.30*
agrimoniin (**10**)	A	44.50	*1.80*	29.87	*0.87*	80.82	*3.29*
davuriciin D2/fragariin A ^1^	D2 ^1^	6.43	*0.19*	2.06	*0.13*	7.64	*0.19*
Sum of ellagitannins	ETs	50.97	*1.51*	31.93	*0.82*	88.42	*2.42*
quercetin-3-*O*-[β-D-xylosyl-(1‴→2″)]-β-D-glucuronoside (**11**)	Q3grx	7.27	*0.28*	6.94	*0.10*	7.21	*0.18*
quercetin-3-*O*-β-D-glucuronoside-7-*O*-β-D-glucoside (**9**)	Q3gr7g	0.73	*0.03*	1.07	*0.03*	0.83	*0.06*
quercetin-3-*O*-β-D-glucuronoside (**12**)	Q3gr	3.39	*0.09*	1.65	*0.02*	1.46	*0.12*
kaempferol-3-*O*-[β-D-xylosyl-(1‴→2″)]-β-D-glucuronoside (**13**)	K3grx	5.09	*0.06*	4.86	*0.22*	5.86	*0.12*
kaempferol-3-*O*-β-D-glucuronoside	K3gr	0.57	*0.01*	0.51	*0.01*	0.42	*0.04*
Sum of flavonols	Fs	17.06	*0.09*	15.02	*0.08*	15.77	*0.10*

^1^ calculated as agrimoniin; SD, standard deviation (n = 6); ETs, a sum of A and D2; Fs, a sum of all above-quantified flavonols.

**Table 3:** Identification of adducts of methylglyoxal (MGO) with quercetin and its glycosides by UPLC-qTOF-MS/MS.

Compound	*t_R_* [min]	[M−H]^−^ [*m*/*z*], Measured	Peak/Adducts
quercetin (Q)	10.01	445.0779	Di-MGO-Q
	10.93; 11.06	373.0567	Mono-MGO-Q (two isomers)
	11.83	301.0355	Q
quercetin-3-*O*-β-D-glucoside *isoquercitrin* (Q3g)	8.78; 8.87; 9.00; 9.12	535.1106	Mono-MGO-Q3g (four isomers)
	9.95	463.0890	Q3g
quercetin-4′-*O*-β-D-glucoside *spiraeoside* (Q4′g)	8.98	607.1279	Di-MGO-Q4′g
	9.61	535.1068	Mono-MGO-Q4′g
	10.67	463.0856	Q4′g
quercetin-3-*O*-β-D-galactoside *hyperoside* (Q3ga)	8.19; 8.28; 8.37	607.1303	Di-MGO-Q3ga (three isomers)
	8.70; 8.79; 8.99; 9.09	535.1099	Mono-MGO-Q3ga (four isomers)
	9.88	463.0894	Q3ga
quercetin-3-*O*-β-D-glucuronoside *miquelianin* (12; Q3gr)	8.21; 8.48; 8.68	621.1082	Di-MGO-Q3gr (three isomers)
	8.83; 9.04; 9.13; 9.31	549.0872	Mono-MGO-Q3gr (four isomers)
	10.20	477.0672	Q3gr
quercetin-3-*O*-[β-D-xylosyl(1‴→2″)]-β-D-glucuronoside *flagarin* (11; Q3grx)	8.09; 8.46; 8.75	753.1536	Mono-MGO-Q3grx (three isomers)
	9.45	609.1108	Q3grx

## Data Availability

Not applicable.

## Supplementary Materials

The following supporting information can be downloaded at: https://www.mdpi.com/article/10.3390/molecules27165293/s1, **part A**: NMR data of compounds **1**, **3**, **4a**, **5**, and **8**–**14**; **part B**: Supplementary Figures S1–S9: Scheme of extraction and isolation of individual polyphenols; UV data of compounds **10** vs. **16**, **5** vs. GA, **11** vs. **12**, **11** vs. **13**; MS spectra of MGO adducts with **12** and **13**; NMR data for **4a**; NMR data for **13**; HSQC details for **10**; Supplementary Table S1: The antiglycation activity of selected flavonols (incl. **12** and **13**) and known inhibitors.

## Sample Availability

Samples of the isolated compounds are available from the authors.

## Appendix A. Details on Isolation of *F. ananassa* cv. Senga Sengana Leaf Constituents

Dry strawberry cv. Senga Sengana leaves (FaSS1, 500 g) were crushed with a hand blender in a water and acetone mixture (1+1, *v*/*v*; 6 L) at room temperature. The resulting suspension was filtered by a slit glass (G0) and concentrated under reduced pressure to give the water residue of the aq. acetone extract, FaSS-A (3 L). After acidification with formic acid (up to 0.5%, *v*/*v*), FaSS-A was subjected to column chromatography (CC) on octadecyl (J.T. Baker, Phillipsburg, NJ, USA). Components of FaSS-A were eluted using water and methanol in a stepwise gradient manner. The separation of compounds was monitored by TLC Si60 (mobile phases **E**, **F**, and **P**) and HPLC-DAD methods. Eluates with similar compositions were pooled and concentrated to give 13 fractions: FaSS-0 to FaSS-XII. FaSS-0 was eluted with 0.5% formic acid (from the FaSS-A application) and vacuum concentrated. The other fractions were eluted with water-methanol and methanol. Fractions FaSS-I to FaSS-VIII were concentrated under reduced pressure, followed by the SPE method on a C18. This procedure was developed and described previously by Fecka et al. [82]. Fractions FaSS-IX to FaSS-XII were subsequently concentrated under reduced pressure and allowed to crystallize or sediment. Vacuum-dried FaSS-0 was fractionated on a Sephadex LH-20 (Cytiva, Marlborough, MA, USA) by eluting the compounds with methanol. Concentrated FaSS-I was further separated on a Si60 chromatography column (∅ 0.040–0.063 mm; Merck) with methanol–chloroform. Fractions FaSS-II to FaSS-IX were subsequently purified over Sephadex LH-20 with methanol-acetone. Subfractions with similar compositions (TLC, HPLC-DAD) were pooled, concentrated under vacuum (40 °C), and allowed to crystallize or dry. The separated individual compounds were recrystallized from methanol or water–methanol (1+1). The separation steps of *F. ananassa* cv. Senga Sengana leaf components are shown in Figure S1. The purified compounds were then subjected to structural (spectroscopic) analysis.

Compound **1** (235 mg) was isolated together with **2** (386 mg) from FaSS-0 on Sephadex LH-20 using methanol; **3** (420 mg), **4** (**4a**, 65 mg) and **5** (464 mg) were obtained from FaSS-I by purification on Si60 with a methanol–chloroform gradient; **6** (195 mg) and **7** (2533 mg) were separated from FaSS-II on Sephadex LH-20 with acetone and methanol–acetone (1+9); **8** (~11 mg) from FaSS-III with methanol–acetone (1+9); **9** (~27 mg) from FaSS-IV with methanol–acetone (1+9); **10** (5817 mg) from FaSS-V with methanol–acetone (2+8); **11** (~330 mg) from FaSS-VI with methanol–acetone (2+8); **12** (216 mg) from FaSS-VII with methanol–acetone (1+9); **13** (~48 mg) from FaSS-VIII with methanol–acetone (2+8); **14** (~25 mg) from FaSS-IX with methanol–acetone (1+9); **15** (~17 mg) from FaSS-X and **16** (245 mg) from FaSS-XI by crystallization with water–methanol (1+1). Retention parameters of isolated compounds together with tentative UHPLC-qTOF-MS/MS identification of non-isolated compounds from FaSS are given in Table 1.

Structures of compounds **1**, **3**–**5**, **7**–**15** were characterized by spectroscopic evidence (UV-VIS, ESI-MS, 1D- and 2D-NMR) and compared with literature. Compounds **2**, **6**, and **16** were compared chromatographically (R*_f_*, t*_R_*,), UV-VIS (*λ*_max_), and ESI-MS (*m*/*z*) with authentic standards. Experimental NMR data are provided in supplementary materials. Compounds **1**–**3** and **5**–**16** were free from other phenolic impurities (purity ≥ 95%, HPLC-DAD). Compound **4a** was accompanied by a dopant of a close derivative.

## Appendix B. Details on Identification of F. ananassa cv. Senga Sengana Leaf Constituents

### Appendix B.1. Hydrolyzable Tannins

A dimeric ellagitannin (**10**), isolated as a beige, amorphous powder from FaSS-V and identified as agrimoniin, was the most abundant compound in Senga Sengana leaves. Agrimoniin was previously obtained from *Agrimonia pilosa* Ledeb. (syn. *A. japonica* (Miq.) Koidz.) and *Potentilla kleiniana* Wight et Arnott. [17,18], as well as from *F. ananassa* Duch. and *F. nipponica* Makino leaves [29], and berries of woodland and garden strawberry [45]. High-resolution MS^1^ and MS^2^ data of **10** with a pseudomolecular ion at *m*/*z* 934.0722 [M−2H]^−2^ (predominant peak; calc. 934.0718 and 1869.1508, [C_82_H_51_O_52_]^−^) are presented in Table 1. Fragmentation of 10 produced ions derived from structures of secondary ellagitannins and low-molecular depsides (i.e., hexahydroxydiphenoyl—HHDP, and dehydrodigalloyl—DHDG; both 302 Da) formed by partial degradation of agrimoniin at *m*/*z*: 1567 [M−302(HHDP)−H]^−^ (laevigatin B/C/F), 1085 [M−784(pedunculagin)−H]^−^ (agrimonic acid A/B lactone), 935 [M−934−H]^−^ (potentillin), 897 [M−936(potentillin)−36(2H_2_O)−H]^−^, 783 [M−1086(agrimonic acid A/B lactone)−H]^−^ or [M−784(pedunculagin)−302(HHDP)−H]^−^ (pedunculagin α/β or laevigatin A lactone), 745 [M−936(potentillin)−152(galloyl)− 36(2H_2_O)−H]^−^, 633 [M−934−302(HHDP)−H]^−^ (galloyl-HHDP-glucose), 613 [M−1086(agrimonic acid A/B lactone)−152(gall)−18(H_2_O)−H]^−^, 481 [M−1086(agrimonic acid A/B lactone)−302(HHDP)−H]^−^, (HHDP-glucose), 463 [M−1086 (agrimonic acid A/B lactone)−302(HHDP)−18(H_2_O)−H]^−^, 319 [DHDG−18(H_2_O)−H]^−^ (mono-lactone of DHDG), 301 [HHDP−36(2H_2_O)−H]^−^ (dilactone of HHDP, ellagic acid), and 275 [HHDP−18(H_2_O)−44(CO_2_)−H]^−^ (decarboxylated monolactone of HHDP). The presence of ellagic acid in the acid hydrolysis products of **10** was also confirmed by TLC (mobile phase **P**).

The 1D- and 2D-NMR experiments of **10** were conducted in acetone-*d6* with D_2_O (1+1, *v*/*v*). Since the agrimoniin molecule has four HHDP groups, one DHDG coupler and two α-D-glucopyranoses (α-Glc*p*), and it is asymmetric [17] in the ^1^H-NMR spectrum, we observed ten signals typical for these phenolic moieties in an aromatic region and two doublets from anomeric protons of two α-Glc*p* (Figure S9), as well as other sugar signals. For the DHDG unit, it was δ: 7.31 (1H, d, J = 2 Hz), 7.17 (1H, s); for two HHDP units δ: 6.62, 6.60, 6.53, 6.47, 6.45, 6.31, 6.29, 6.28 (each 1H s); for two α-Glcp anomers δ: 6.49, 6.42 (each 1H, d, J = 3.5 Hz) (α-Glc*p*^1^ and α-Glc*p*^2^, H-1,1′), and for other α-Glc*p* protons δ: 5.39, 5.28 (each 1H, t, J = 9.5 Hz, α-Glc*p* H-3,3′), 5.26, 5.22 (each 1H, dd, α-Glc*p* H-2,2′), 5.14, 4.86 (each 1H, t, J = 13.2 Hz, α-Glc*p* H-6^2^,6^2^′), 5.02, 4.93 (1H, t, J = 9.5 Hz, α-Glc*p* H-4,4′), 4.50, 3.88 (1H, dd, J = 9.5, 6.3 Hz, α-Glc*p* H-5,5′), 3.75, 3.40 (1H, d, J = 13.2 Hz, α-Glc*p* H-6^1^,6^1^′). ^13^C-NMR (and HSQC indirectly) of **10** showed ten ester-forming carboxylic groups δ: 169.78, 169.54, 169.45, 168.98, 168.68, 168.35, 168.29, 165.56, 164.59, 164.19 ppm. The following anomeric α-Glc*p* carbons were also HSQC-correlated with respective protons of molecule **10**: H-1/90.79 ppm and H-1′/90.44 ppm. All carbon signals of agrimoniin are reported in Part A of Supplementary Materials.

Compound **7** isolated from FaSS-II as a beige, amorphous powder with a pseudomolecular ion at *m*/*z* 783.0676 (calc. 783.0686, [C_34_H_23_O_22_]^−^) and fragmentation typical for hexahydroxydiphenoyl esters (481, 301, 275) was tentatively identified as a monomeric ellagitannin named pedunculagin. ^1^H-NMR analysis of **7** confirmed this assumption [16].

Compound **3** and **5** (from FaSS-I) with identical pseudomolecular ions at *m*/*z* 343.0669 [M−H]^−^ (predominant, calc. 343.0671, [C_14_H_15_O_10_]^−^) produced fragments deriving from both quinic (191 [QA−H]^−^, 173 [QA−18(H_2_O)−H]^−^) and gallic acids (169 [GA−H]^−^, 125 [GA−44(CO_2_)−H]^−^). A galloyl moiety was also detected after acid hydrolysis of 3 and 5 (TLC, mobile phase **P**). Those monogalloylquinic acid regioisomers showed UV-VIS spectra with the 2nd absorption maximum at 273 nm, typical for galloyl esters. The NMR data of **3** and **5** are summarized in Supplementary Materials. Monogalloylquinic acids **3** and **5** (classified in gallotannins) were therefore identified as 3- and 5-*O*-galloylquinic acid, respectively [14,15].

### Appendix B.2. Proanthocyanidins and Flavan-3-Ols

Condensed tannins were minor *F. ananassa* cv. Senga Sengana components. Several B-type proanthocyanidins (five dimers, three trimers, and one tetramer) were detected together with monomeric catechin by UHPLC-qTOF-MS/MS (*m*/*z* 289.0719, 577.1346, 865.1981, 1153.2610). Catechin (**6**) was also isolated as a fine white powder from fraction FaSS-II. Chromatographic and spectroscopic data (R_f_, t_R_, UV-VIS, ESI-MS) of 6 were consistent with the authentic standard of this compound. Procyanidins B3 and C2 as well as other oligomeric flavan-3-ol derivatives of both catechin and epicatechin were recognized based on UV-VIS profiles and MS fragmentations. Three distinct fragmentation pathways were observed for these proanthocyanidins: loss of neutral molecules by HRF (heterocyclic ring fission, −126 Da), RDA (retro-Diels-Alder fission, −152 Da), and QM (quinone methide fission, Δ 2H) [87,88]. In particular, fragment ions released during C-C bond fission by QM were diagnostically relevant in the analysis of B-type proanthocyanidins, e.g., for procyanidin B3 these ions were at 287 and 289, for procyanidin C2 at 575, 577, 287 and 289 (Table 1). Catechin and procyanidins B3 and B6 were formerly isolated from *F. ananassa* cv. Reikov roots [26].

### Appendix B.3. Flavonoids

Three diglycosides, i.e., **9** (from FaSS-IV), **11** (from FaSS-VI), and **13** (from FaSS-VII), as well as four monoglycosides, i.e., **8** (FaSS-III), **12** (FaSS-VII), **14** (FaSS-IX) and **15** (FaSS-X), were isolated from FaSS-A. Glucuronic acid, glucose, and xylose were detected in their glycone parts after acid hydrolysis. The sugar-aglycone connections were based on HMBC/NOESY, as well as by other experiments (Section 4.6). Some of the noted flavonoids, including flavonol diglycosides (MW 594, 610, and 640 Da), were described previously in leaves of *F. ananassa* cv. Polka [27], in flowers of cv. Jonsok [28], and in pseudo-fruits of the Japanese cultivar Tochiotome [21]. Nevertheless, their complete structure has not been established or finely defined.

Compound **9** (yellow crystalline powder) exhibited a pseudomolecular ion at *m*/*z* 639.1200 [M−H]^−^ (calc. 639.1203 for [C_27_H_27_O_18_]^−^) that is relevant to hexoside-hexuronide of quercetin-like aglycone (ions at 301, 179 and 151). The MS^2^ spectrum revealed the presence of a fragment ion at *m*/*z* 463 [M−176−H]^−^ corresponding to partially degraded diglycoside after the loss of hexuronic acid and suggested that sugars must be attached independently. Both glucose and glucuronic acid were released by acidic hydrolysis, confirmed by TLC (mobile phase **S**). The 1D and 2D-NMR experiments on **9** were conducted in DMSO-*d6*. All data were similar to those presented by Felser and Schimmer [20]. Briefly, the typical quercetin pattern of proton signals was not affected by glycosylation in flavonol ring B. A glucose (β-Glc*p*) moiety was connected at position C-7 of aglycone, which was proved by NOESY (H-1‴↔H-8). The glucuronoside moiety was attached at position C-3 (H-1 of β-GlcAp showed HMBC correlation with C-3). Finally, compound **9** was labelled as quercetin-3-*O*-β-glucuronoside-7-*O*-β-glucoside.

The MS spectrum of compound **8** (beige powder) with ions at *m*/*z* 465.1035 [M−H]^−^ (calc. 465.1039, [C_21_H_21_O_12_]^−^), 303 [M−162−H]^−^ and 285 [303−18−H]^−^ was relevant to hexoside of dihydroquercetin (taxifolin). The loss of a water molecule from the aglycone during fragmentation of **8** demonstrated unambiguously its flavanonol structure and indicated the likely sugar substitution position at C-3. The NMR analysis was conducted in CD_3_OD to compare the results with the reference. All spectroscopic data of **8** were clearly similar to those presented by Sakushima et al. [19] and different from those of Pan and Lundgren [47], but the spectrum revealed a number of overlapping isomer signals. The typical dihydroquercetin pattern of proton signals was not affected by glycosylation in flavonoid rings A and B. Glycosylation at position C-3 was based on shifts of H-2 and H-3: 5.24 (d, J = 9.7 Hz) and 4.93 ppm (d, J = 9.8 Hz), in comparison to unsubstituted 2,3-dihydroquercetin. The absolute configuration at C-2 and C-3 of the main compound in the mixture was provisionally assigned by comparison with Sakushima et al. [19] to be 3-*O*-β-glucoside of (2*R*,3*R*)-taxifolin (dihydroquercetin). That compound was also found in Tochiotome strawberries by Abe et al. [21]. Other taxifolin pentosides and hexosides were also observed in LC-MS as minor components (Table 1). For example, taxifolin-3-*O*-α-arabinoside was isolated previously from roots of *F. ananassa* cv. Reikov by Ishimaru et al. [26].

Based on spectroscopic evidence, flavonoids **12**, **14** and **15** were identified as quercetin-3-*O*-β-glucuronoside (syn. quercetin-3-*O*-β-glucuronide or miquelianin, *m*/*z* 477.0672), isorhamnetin-3-*O*-β-glucuronoside (3′-*O*-methylquercetin-3-*O*-β-glucuronoside, 491.0806), and tiliroside (kaempferol-3-*O*-β-(6″-*O*-*p*-coumaroyl) glucoside, 593.1299; a pair of cis/trans isomers), respectively [22,23,24,38,39,40,41]. Using UHPLC-qTOF-MS/MS, the following flavonoids were additionally detected: quercetin-3-*O*-β-(glucosyl)glucuronoside (tentatively, based on MS and MS^2^)—*m*/*z* 639.1200 [M−H]^−^, quercetin-3-*O*-β-glucoside (isoquercitrin, confirmed with standard)—463.0878 [M−H]^−^, kaempferol-3-*O*-β-glucuronoside (confirmed with standard)—461.0724 [M−H]^−^ and kaempferol-3-*O*-β-glucoside (astragalin, confirmed with standard)—447.0929 [M−H]^−^. A different fragmentation pattern than for quercetin-3-*O*-β-glucuronoside-7-*O*-β-glucoside characterized quercetin-3-*O*-β-(glucosyl)glucuronoside. Its glycone moiety, structurally similar to 3-*O*-[β-xylosyl(1‴→2″)]-β-glucuronosides of quercetin and kaempferol, was uncoupled as a whole disaccharide chain [M−338−H]^−^, in applied conditions of ESI-qTOF-MS/MS.

### Appendix B.4. Phenolic and Carboxylic Acids

UHPLC-qTOF-MS/MS analysis of *F. ananassa* cv. Senga Sengana leaf extracts showed intense peaks of several phenolic acid derivatives, as well as carboxylic acids. Compound **1** was isolated from FaSS-0 as a white crystalline powder with *λ*_max_ at 315 nm, and pseudomolecular ions at *m*/*z* 182.9934 [M−H]^−^ (predominant, calc. 182.9935, [C_7_H_3_O_6_]^−^) and 366.9940 [2M−H]^−^. Its fragmentation-derived ions suggested a subsequent neutral loss of carbon dioxide and carbon oxide: 139 [M−44/CO_2_−H]^−^ and 111 [M−44/CO_2_−28/CO−H]^−^. The ^1^H- and ^13^C-NMR (in CD_3_OD) data of **1** were consistent with results published by Wilkes and Glasl [25]. Thus, **1** was identified as 2-pyrone-4,6-dicarboxylic acid (PDC).
