# Supplementary material for: Fragaria × ananassa cv. Senga Sengana Leaf: An Agricultural Waste with Antiglycation Potential and High Content of Ellagitannins, Flavonols, and 2-Pyrone-4,6-dicarboxylic Acid
Source: Molecules. 2022 Aug 19;27(16):5293. doi: 10.3390/molecules27165293 (PMC9415522; doi:10.3390/molecules27165293)
Supplement: Supplementary file 1 [file molecules-27-05293-s001.zip › molecules-1868113-supplementary.pdf]

Supplementary Materials to

Fragaria × ananassa cv. Senga Sengana leaf: An agricultural waste with antiglycation potential and high content of ellagitannins, flavonols, and 2-pyrone-4,6-dicarboxylic acid

Izabela Fecka [1], Katarzyna Bednarska [1] and Maciej Włodarczyk [1,*]

[1] Department of Pharmacognosy and Herbal Medicines, Faculty of Pharmacy, Wroclaw Medical University, Borowska 211a, 50-556 Wroclaw, Poland

* Correspondence: maciej.wlodarczyk@umw.edu.pl; Tel.: +48-71-78-40-223

A. NMR data for compounds 1, 3, 4a, 5, 8-14:

***Rosaceae* 2-pyrone-4,6-dicarboxylic acid (PDC, 1)**, creamy powder; ^{1}H-NMR (300 MHz, CD_3OD): δ 7.43 (1H, d, J=1.5Hz, H-3), 6.75 (1H, d, J=1.5 Hz, H-5). ^{13}C-NMR (75 MHz, CD_3OD): δ 162.20 (s, C-2), 107.81 (s, C-3), 155.28 (s, C-6), 168.73 (s, C-7); 163.89 (s, C-8).

3-*O*-Galloylquinic acid (3-GQA, 3), light beige powder; ^{1}H-NMR (300 MHz, CD_3OD): δ 7.07 (s, 2H, H-2' & H-6'), 5.31 (m, 1H, H-3), 4.10 (m, 1H, H-5), 3.63-3.49 (m, 1H, H-4), 2.13+1.86 (m, 4H, H-2 & H-6); ^{13}C NMR (75 MHz, CD_3OD): δ 167.47 (C-7'), 146.19 (C-3' & C-5'), 139.79 (C-4'), 109.92 (C-2' & C-6'), 121.20 (C-1'), 76.00 (C-1), 72.25 (C-3), 71.55 (C-5), 64.20 (C-4), 42.02 (C-2 & C-6).

Protocatechuoyl xylose (4a), white amorphous powder; ^{1}H NMR (300 MHz, DMSO-*d6*) δ 7.37 (1H, d, J=3.1 Hz, H-2), 6.86 (1H, dd, J=8.7, 3.1 Hz, H-6), 6.56 (1H, d, J=8.7 Hz, H-5), 4.57 (1H, d, J=7.1 Hz, H-1′), 3.73 (1H, dd, J=11.2, 5.1 Hz, 1H, H-5′a), 3.34 (1H, d, J=10.6 Hz, H-4′), 3.18 (2H, m, H-2′ & H-3′), 3.12 (1H, t, J=11.3 Hz, H-5′b); ^{13}C NMR (75 MHz, DMSO-*d6*) δ 171.38 (C-7), 157.66 (C-4), 147.99 (C-3), 121.52 (C-6), 120.31 (C-1), 118.30 (C-2), 115.81 (C-5), 103.12 (C-1′), 76.48 (C-3′), 73.20 (C-2′), 69.46 (C-4′), 65.68 (C-5′).

5-*O*-Galloylquinic acid (5-GQA, 5), light beige powder; ^{1}H-NMR (300 MHz, CD_3OD): δ 7.02 (s, 2H, H-2′ & H-6′), 5.39 (m, 1H, H-5), 4.19 (m, 1H, H-3), 3.86-3.75 (m, 1H, H-4), 2.22+2.05 (m, 4H, H-2 & H-6); ^{13}C-NMR (75 MHz, CD_3OD): δ 175.27 (C-7), 167.69 (C-7′), 146.27 (C-3′ & C-5′), 139.72 (C-4′), 110.05 (C-2′ & C-6′), 121.49 (C-1′), 76.00 (C-1), 71.98 (C-5), 70.67 (C-3), 61.82 (C-4), 38.11 (C-2 & C-6).

(2*R*,3*R*)-taxifolin-3-*O*-β-glucoside (8), brown powder; ^{1}HNMR (300 MHz, CD_3OD): δ 6.95 (1H, d, J=2.0 Hz; H-2′), 6.91 (1H, s; H-6), 6.90 (1H, s; H-8), 6.83 (1H, dd, J=2.8, 8.1 Hz; H-6′), 6.78 (1H, d, J=8.1 Hz; H-5′), 5.24 (1H, d, J=9.8 Hz; H-2), 4.92 (1H, d, J=9.8 Hz; H-3).

Quercetin-3-*O*-β-glucuronoside-7-*O*-β-glucoside (9), yellow powder; ^{1}HNMR (300 MHz, DMSO-*d6*): δ 7.61 (1H, d, J=1.7 Hz; H-2′), 7.58 (1H, dd, J=8.3, 2.0 Hz; H-6′), 6.85 (1H, d, J=8.5 Hz; H-5′), 6.76 (1H, d, J=2.1 Hz; H-8), 6.45 (1H, d, J=2.1 Hz; H-6), 5.50 (1H, d, J=7.0 Hz; H-1″), 5.09 (1H, d, J=7.2 Hz; H-1‴), 3.44 (m; H-5‴), 3.29 (m; H-3‴), 3.29 (m; H-3″), 3.57 (m; H-5″), 3.29 (m; H-2″), 3.29 (m; H-2‴), 3.39 (m; H-4″), 3.18 (m; H-4‴), 3.70 (m; H-6‴a) , 3.47 (m; H-6‴b); ^{13}C NMR (75 MHz, DMSO-*d6*) δ 177.36 (C-4), 170.33 (C-6″), 162.93 (C-7), 160.81 (C-5), 158.35 (C-9), 155.91 (C-2), 148.8 (C-4′), 144.93 (C-3′), 133.38 (C-3), 121.72 (C-6′), 120.72 (C-1′), 116.39 (C-2′), 115.19 (C-5′), 105.52 (C-10), 101.07 (C-1″), 99.79 (C-1‴), 99.42 (C-6), 94.49 (C-8), 77.17 (C-5‴), 76.4 (C-3‴), 75.9 (C-3″), 75.9 (C-5″), 73.78 (C-2″), 73.11 (C-2‴), 71.34 (C-4″), 69.55 (C-4‴), 60.61 (C-6‴).

Agrimoniin (10), yellow amorphous powder; ^{1}H NMR (500 MHz, acetone-*d6*+D_2O, 1:1): δ 7.31 (1H, d, J=2.0 Hz), 7.17 (1H, s) (DHDG/m-GOG), 6.62, 6.60, 6.53, 6.47, 6.45, 6.31, 6.29, 6.28 (1H each, s) (4 × HHDP), 6.49, 6.42 (1H each, d, J=3,5 Hz) (anomers *α*-Glc*p*1 and *α*-Glc*p*2, H-1,1′), 5.39, 5.28 (1H each, t, J=9.5 Hz, Glc*p* H-3,3′), 5.26, 5.22 (1H each, dd, Glc*p* H-2,2′), 5.14, 4.86 (1H each, t, J=13.2 Hz, Glc*p* H-62,62′), 5.02, 4.93 (1H, t, J=9.5 Hz, Glc*p* H-4,4′), 4.50, 3.88 (1H, dd, J=9.5, 6.3 Hz, Glc*p* H-5,5′), 3.75, 3.40 (1H, d, J=13.2 Hz, Glc*p* H-61,61′); ^{13}C NMR (125 MHz, acetone-*d6*+D_2O, 1:1): δ 169.78, 169.54, 169.45, 168.98, 168.68, 168.35, 168.29, 165.56, 164.59, 164.19 (10 × –COOR), 147.42, 146.27 (1C each), 144.84, 144.75, 144.12, 144.01, 143.79, (3C each), 142.98 (2C), 140.90, 140.18, 139.29 (1C each), 136.58, 136.38, 136.34 (1C each), 136.14-135.94 (6C), 125.68, 125.52, 125.58, 125.42, 125.29, 125.21, 124.91, 124.68, 124.03 (1C each), 119.34, 115.64, 115.35, 115.23, 114.99, 114.18, 114.16, 114.13, 113.93, 113.91, 112.06, 109.70, 108.43, 107.90, 107.85, 107.62, 107.55, 107.15, 106.90, 106.72 (total 60C, DHDG/m-GOG & 4 × HHDP), 90.79 (Glc*p* C-1), 90.44 (Glc*p* C-1′), 75.32 (2C, Glc*p* C-3 & 3′), 73.72 (Glc*p* C-2), 73.62 (Glc*p* C-2′), 70.61 (Glc*p* C-5), 70.40 (Glc*p* C-5′), 68.79 (Glc*p* C-4), 68.45 (Glc*p* C-4′), 63.06 (Glc*p* C-6), 63.01 (Glc*p* C-6′).

Quercetin-3-*O*-[β-xylosyl(1‴→2″)]-β-glucuronoside (flagarin, 11), data consistent with (Abe et al., 2018). Yellow amorphous powder; ^{1}HNMR (300 MHz, DMSO-*d6*): δ 7.71 (1H, d, J=2.2 Hz, H-6′), 7.59 (1H, d, J=8.3, 2.1 Hz H-2′), 6.83 (1H, d, J=8.6 Hz, H-5′), 6.37 (1H, d, J=2.0 Hz, H-8), 6.16 (1H, d, J=2.0 Hz, H-6), 5.70 (1H, d, J=7.6 Hz, H-1″), 4.58 (1H, d, J=6.9 Hz, H-1‴), 3.67 (H-5‴a), 3.51 (H-2″), 3.33 (H-2‴), 3.02 (H-5‴b); other sugar's protons visible as multiplet; ^{13}C NMR (75 MHz, DMSO-*d6*) δ 177.43 (C-4), 171.46 (C-6″), 164.21 (C-7), 161.20 (C-5), 160.03 (C-2), 157.15 (C-9), 148.58 (C-4′), 144.90 (C-3′), 133.27 (C-3), 121.87 (C-1′), 121.43 (C-6′), 116.43 (C-2′), 115.13 (C-5′), 104.29 (C-1‴), 103.81 (C-10), 98.48 (C-6), 98.15 (C-1″), 93.31 (C-8), 81.37 (C-2″), 76.49* (C-4″), 76.45* (C-3″), 76.26* (C-3‴), 74.30 (C-2‴), 73.38 (C-5″), 71.43 (C-4‴), 65.25 (C-5‴). *overlapping shifts can be exchanged.

Quercetin-3-*O*-β-glucuronoside (12), data consistent with (Ossipov et al., 1995). Yellow crystalline powder; ^{1}HNMR (300 MHz, DMSO-*d6*): δ 7.61 (1H, d, J=2.1 Hz, H-2′), 7.57 (1H, dd, J=8.3, 2.1 Hz H-6′), 6.84 (1H, d, J=8.3 Hz, H-5′), 6.41 (1H, d, J=2.0 Hz, H-8), 6.21 (1H, d, J=2.0 Hz, H-6), 5.49 (1H, d, J=7.3 Hz, H-1″), 3.28 (m; H-3″), 3.57 (d, J=9.5 Hz, 1H; H-5″), 3.29 (m; H-2″), 3.38 (m; H-4″); ^{13}C NMR (75 MHz, DMSO-*d6*) δ 177.36 (C-4), 169.86 (C-6″), 164.41 (C-7), 161.26 (C-5), 156.33 (C-9), 156.22 (C-2), 148.71 (C-4′), 145.04 (C-3′), 133.04 (C-3), 121.8 (C-6′), 120.95 (C-1′), 116.77 (C-2′), 116.3 (C-5′), 103.96 (C-10), 101.29 (C-1″), 98.9 (C-6), 93.7 (C-8), 75.96 (C-3″), 75.43 (C-5″), 74.1 (C-2″), 71.39 (C-4″).

Kaempferol-3-*O*-[β-xylosyl(1‴→2″)]-β-glucuronoside (13), previously unreported, data compared with (Abe et al., 2018). Yellowish crystalline powder. ^{1}H NMR (300 MHz, DMSO-*d6*) δ 8.07 (2H, d, J=8.9 Hz, H-2′ & H-6′), 6.88 (2H, d, J=8.6 Hz, H-3′ & H-5′), 6.37 (1H, d, J=2.1 Hz, H-8), 6.14 (1H, d, J=2.1 Hz, H-6), 5.73 (1H, d, J=7.3 Hz, H-1″), 4.61 (1H, d, J=6.9 Hz, H-1‴), 3.72 (H-5‴a), 3.44 (H-2″), 3.33 (H-2‴), 3.05 (H-5‴b); ^{13}C NMR (75 MHz, DMSO-*d6*) δ 177.43 (C-4), 171.46 (C-6″), 164.29 (C-7), 161.15 (C-5), 160.09 (C-4′), 160.03 (C-2), 156.28 (C-9), 132.68 (C-3), 131.02 (C-2′, C-6′), 125.09 (C-1′), 115.20 (C-3′, C-5′), 104.59 (C-1‴), 103.81 (C-10), 98.70 (C-6), 97.94 (C-1″), 93.69 (C-8), 81.60 (C-2″), 76.45* (C-4″), 76.42* (C-3″), 76.21* (C-3‴), 74.36 (C-2‴), 73.27 (C-5″), 71.49 (C-4‴), 65.38 (C-5‴). *overlapping shifts can be exchanged.

Isorhamnetin-3-*O*-β-glucuronoside (3′-*O*-methylquercetin 3-*O*-β-glucuronoside; 14), data consistent with (Im et al., 2017). Yellow crystalline powder; ^{1}H NMR (300 MHz, DMSO-*d6*) δ 8.21 (1H, d, J=2.0 Hz, H-2′), 7.52 (1H, dd, J=8.1, 2.0 Hz, H-6′), 6.84 (1H, d, J=8.1 Hz, H-5′), 6.41 (1H, d, J= 2.0 Hz, H-8), 6.19 (1H, d, J= 2.0 Hz, H-6) 5.38 (1H, d, J=7.6 Hz, H-1″), 3.57 (3H, s, OCH3), 3.59-3.44 (m, 4H, H-2″, 3″,4″, 5″); ^{13}C NMR (75 MHz, DMSO-*d6*) δ 176.72 (C-4), 168.88 (C-6″), 164.19 (C-7), 161.05 (C-5), 156.34 (C-9), 156.29 (C-2), 148.55 (C-4′), 144.76 (C-3′), 121.55 (C-6′), 120.81 (C-1′), 115.86 (C-2′), 114.96 (C-5′), 103.85 (C-10), 101.27 (C-1″), 98.74 (C-6), 93.4 (C-8), 75.44 (C-3″), 71.21 (C-4″).

B. Supplementary Figures and Tables:

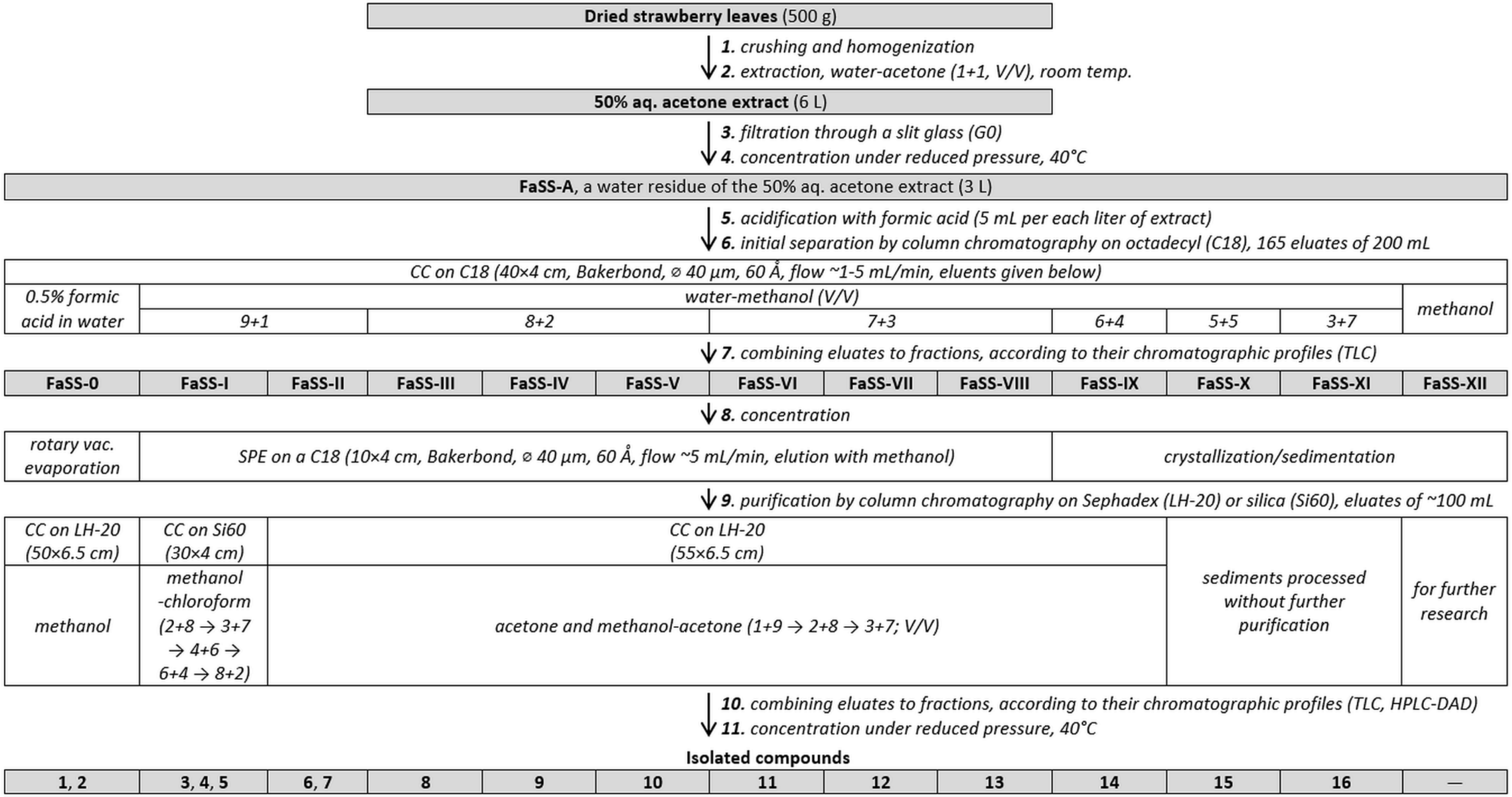

Figure S1. Scheme of extraction and isolation of individual polyphenols from *Fragaria × ananassa* cv. Senga Sengana leaves.

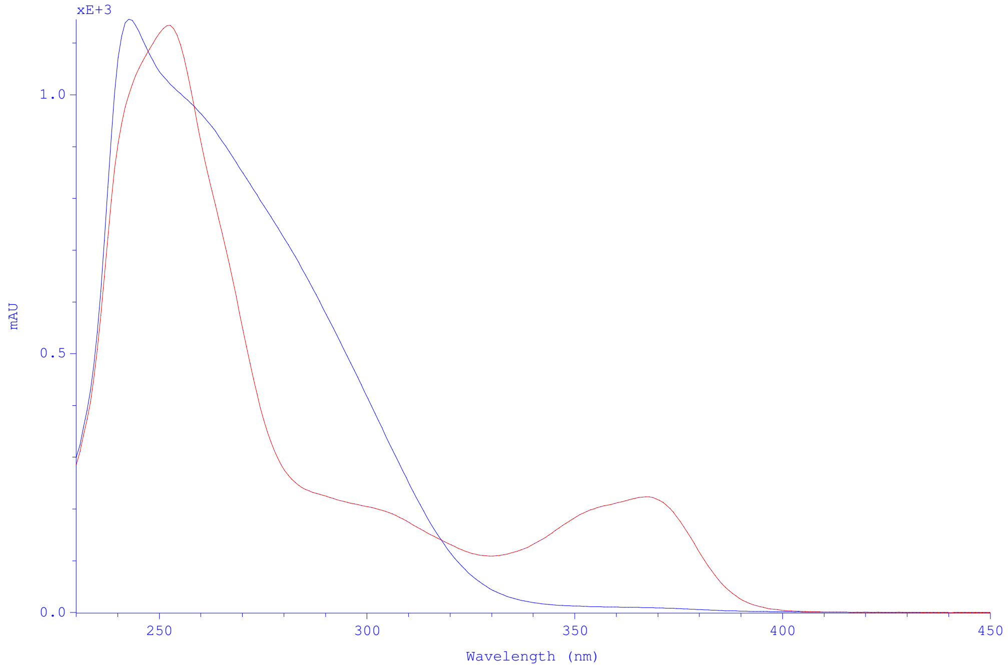

Figure S2. UV-VIS spectra of agrimoniin (**10**, blue line) and ellagic acid (**16**, red line).

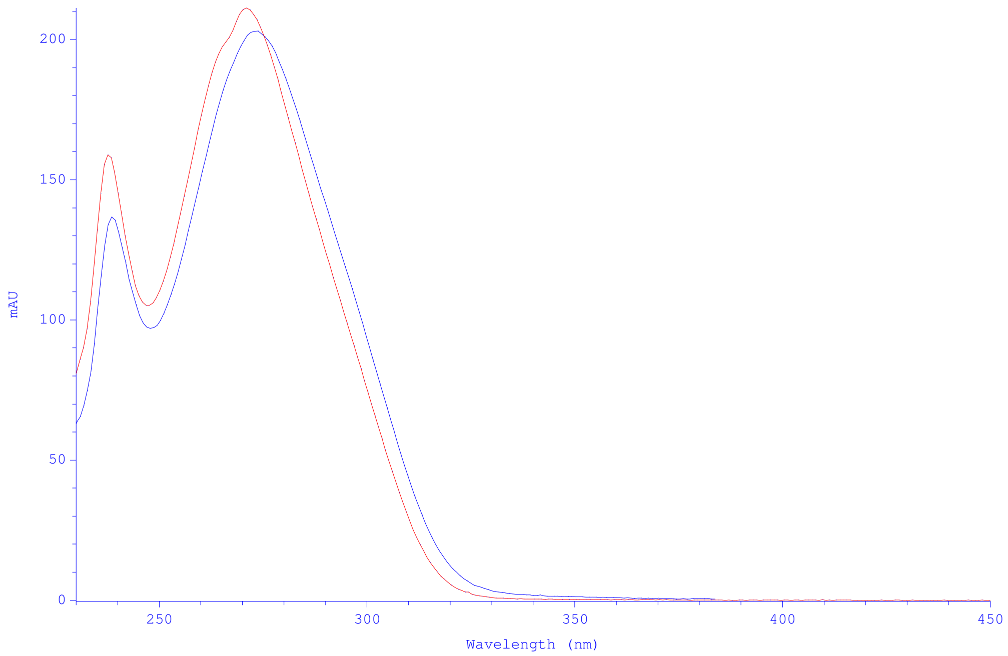

Figure S3. UV-VIS spectra of 5-*O*-galloylquinic acid (**5**, blue line) and gallic acid (red line).

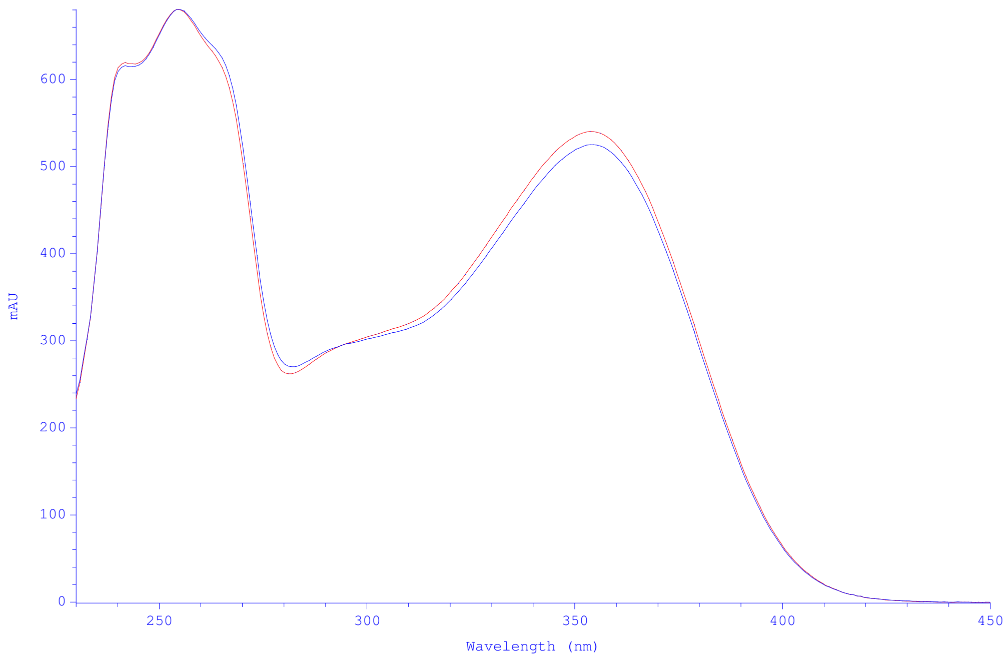

Figure S4. UV-VIS spectra of quercetin-3-*O*-[β-xylosyl(1‴→2″)]-β-glucuronoside (**11**, blue line) and quercetin-3-*O*-β-glucuronoside (**12**, red line).

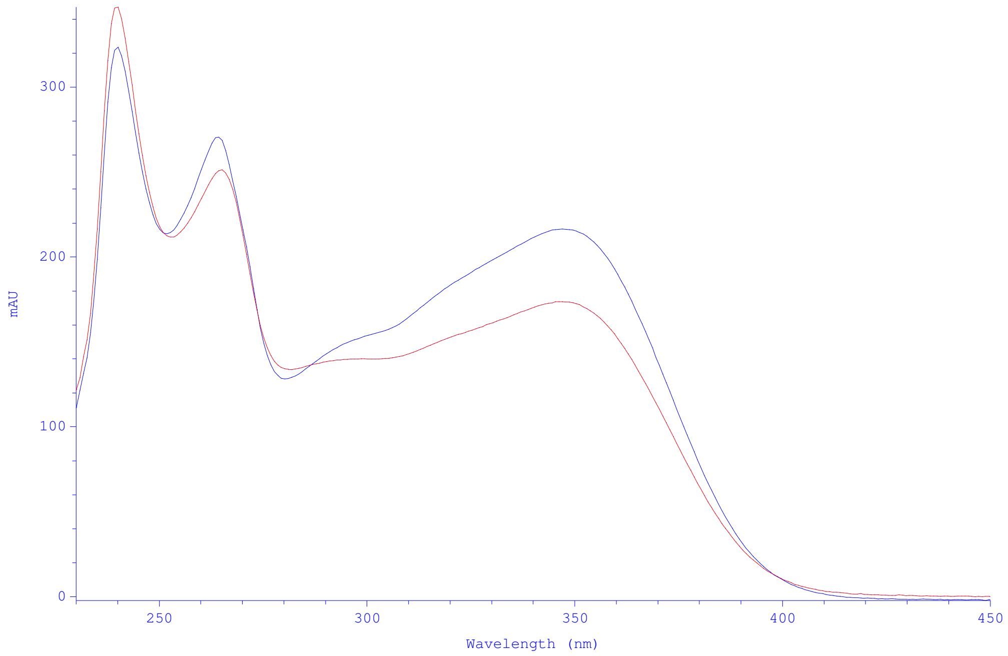

Figure S5. UV-VIS spectra of quercetin-3-*O*-[β-xylosyl(1‴→2″)]-β-glucuronoside (**11**, blue line) and kaempferol-3-*O*-[β-xylosyl(1‴→2″)]-β-glucuronoside (**13**, red line).

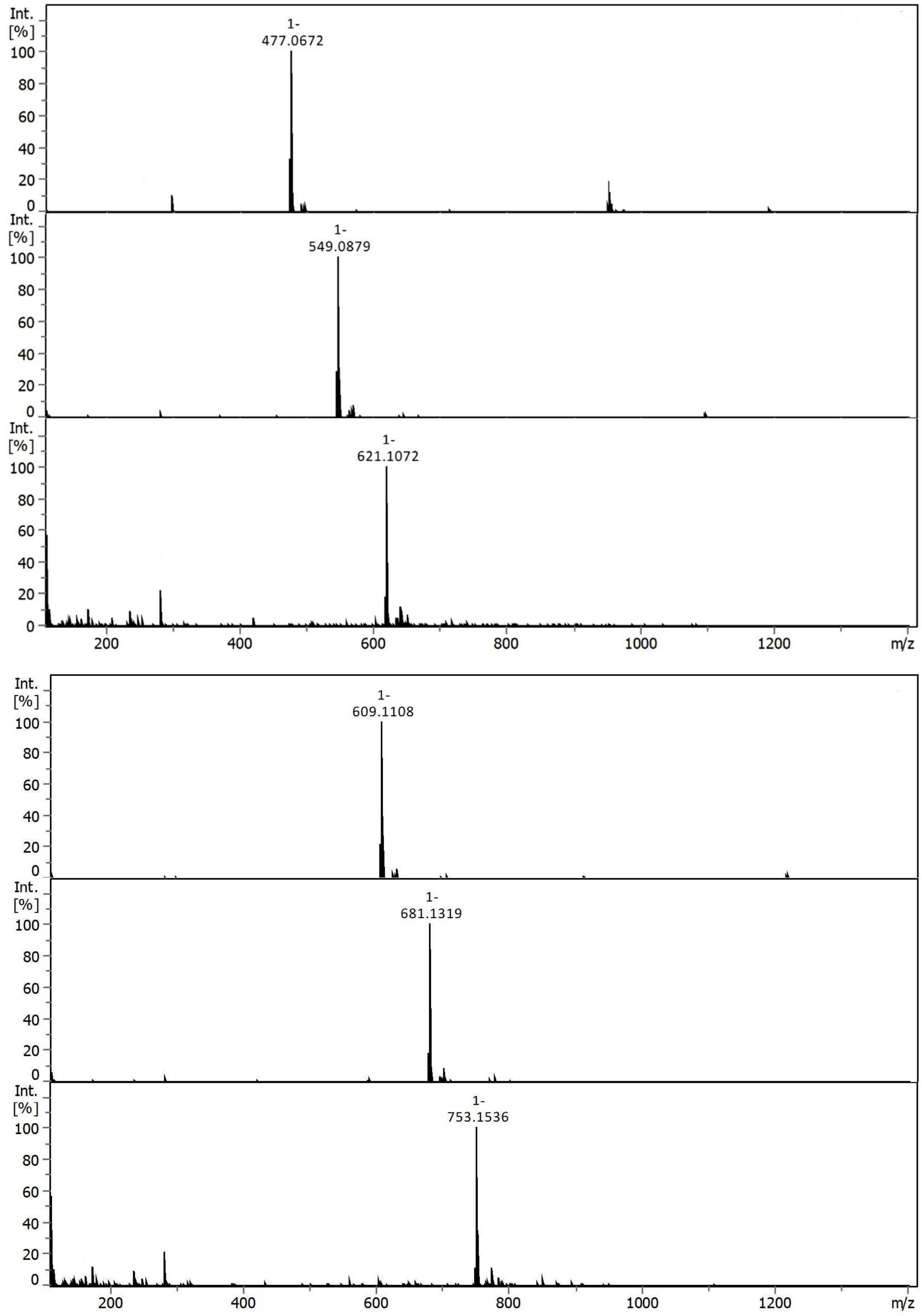

Figure S6. Methylglyoxal adducts with quercetin-3-*O*-β-glucuronoside (above; **12**, miquelianin, Q3gr) and quercetin-3-*O*-[β-xylosyl(1‴→2″)]-β-glucuronoside (below; **11**, flagarin, Q3grx).

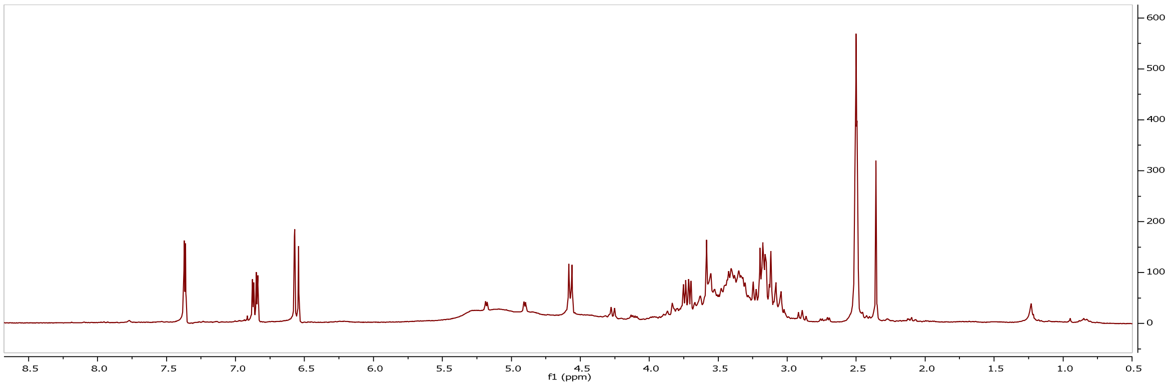

Figure S7a. ^{1}H spectrum of protocatechuoyl xylose (**4a**); 300MHz, in DMSO-*d*6).

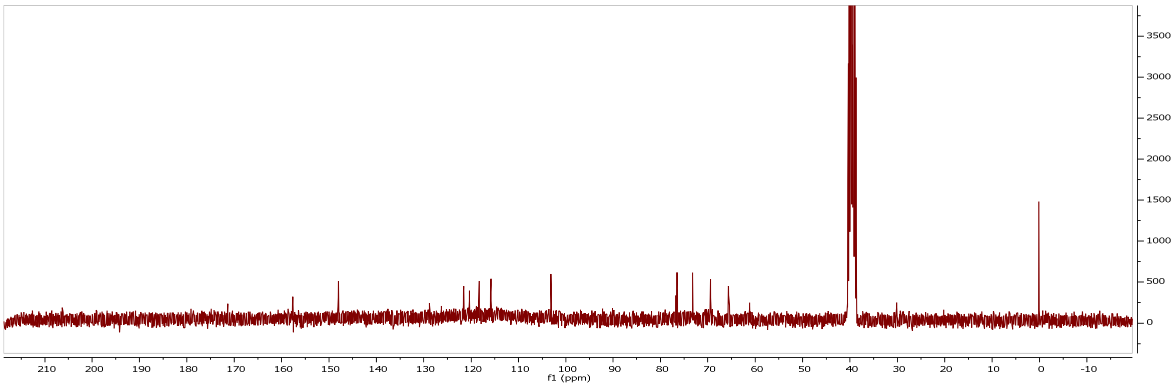

Figure S7b. ^{13}C spectrum of protocatechuoyl xylose (**4a**); 75MHz, in DMSO-*d*6).

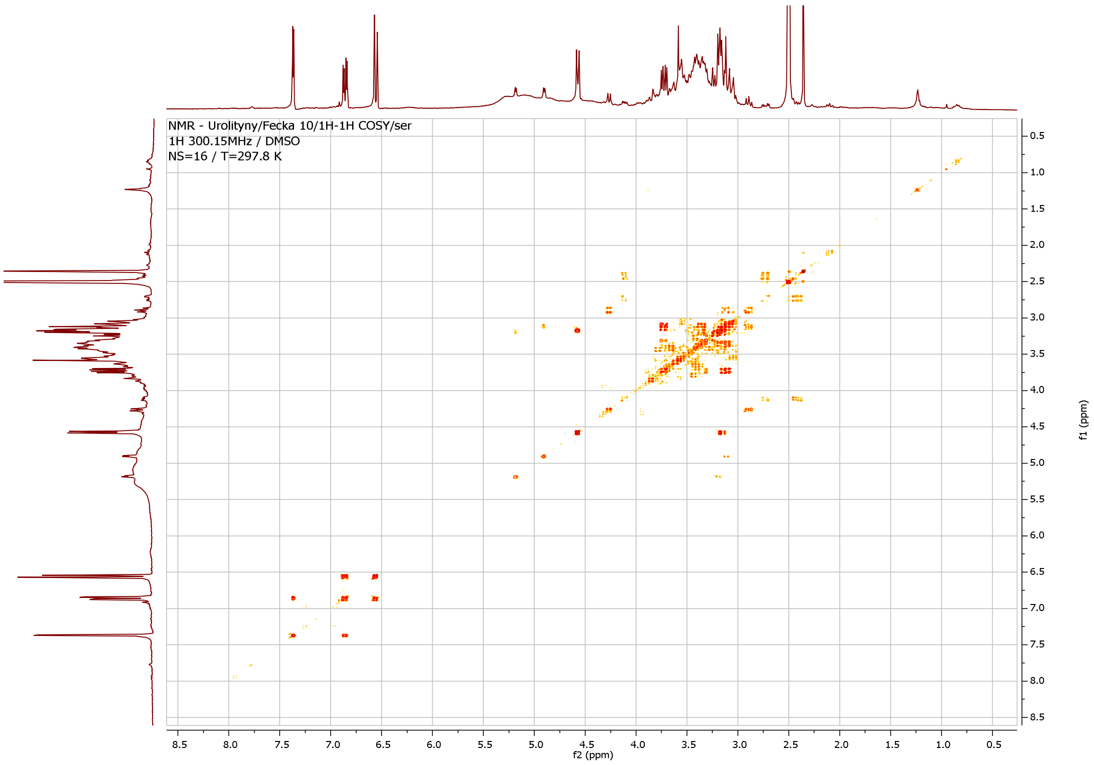

Figure S7c. ^{1}H-^{1}H COSY spectrum of protocatechuoyl xylose (**4a**); 300MHz, in DMSO-*d*6).

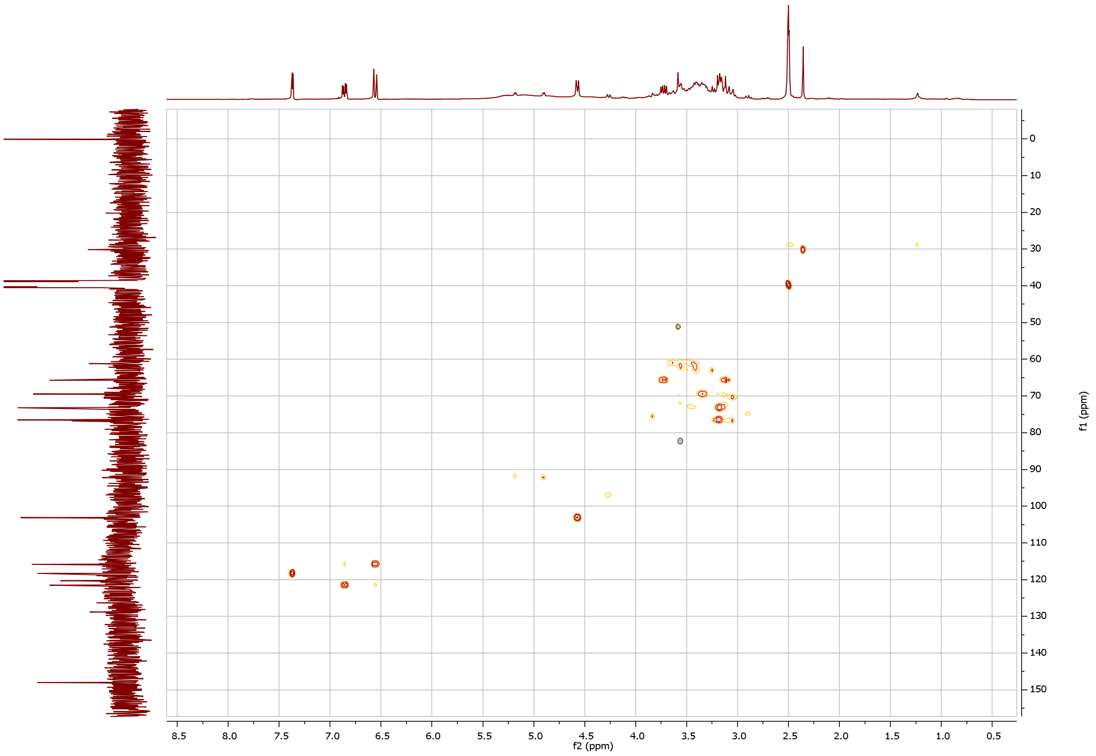

Figure S7d. ^{1}H-^{13}C HSQC spectrum of protocatechuoyl xylose (**4a**) (DMSO-d_6).

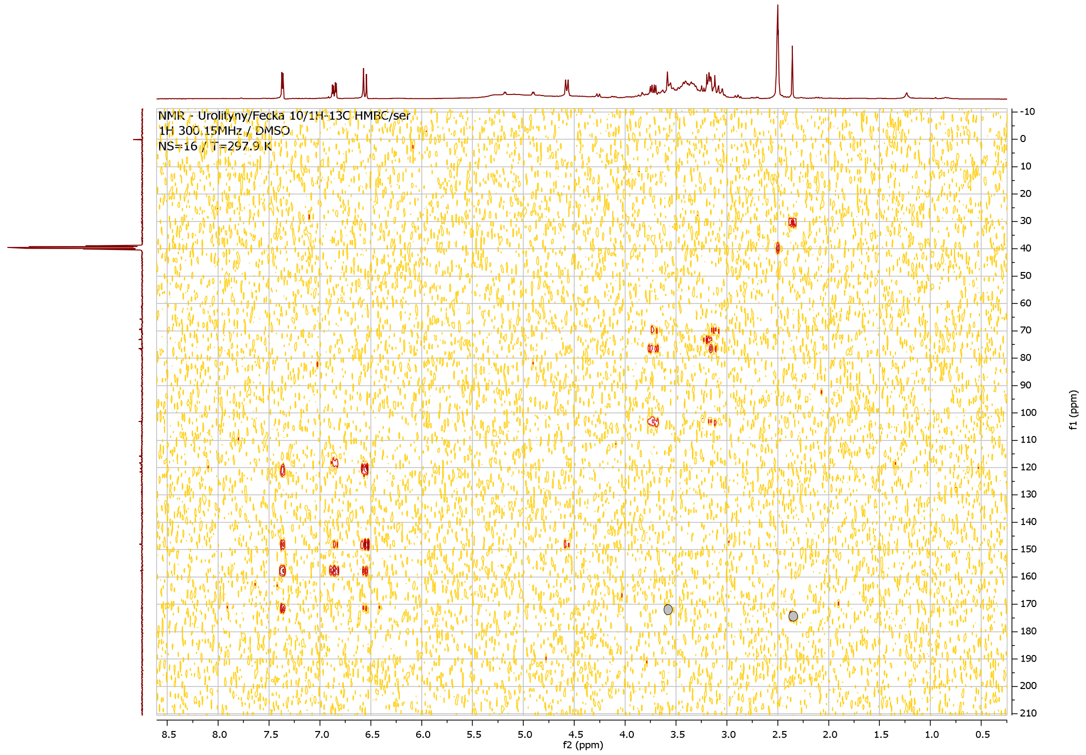

Figure S7e. ^{1}H-^{13}C HMBC spectrum of protocatechuoyl xylose (**4a**) (DMSO-d_6).

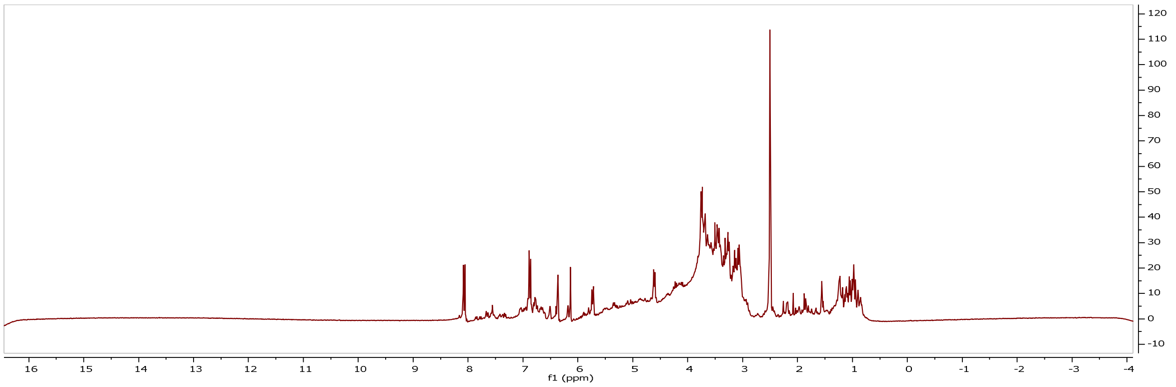

Figure S8a. ^{1}H spectrum of kaempferol-3-O-[β-xylosyl(1‴→2″)]-β-glucuronoside (**13**); 300MHz, in DMSO-d_6).

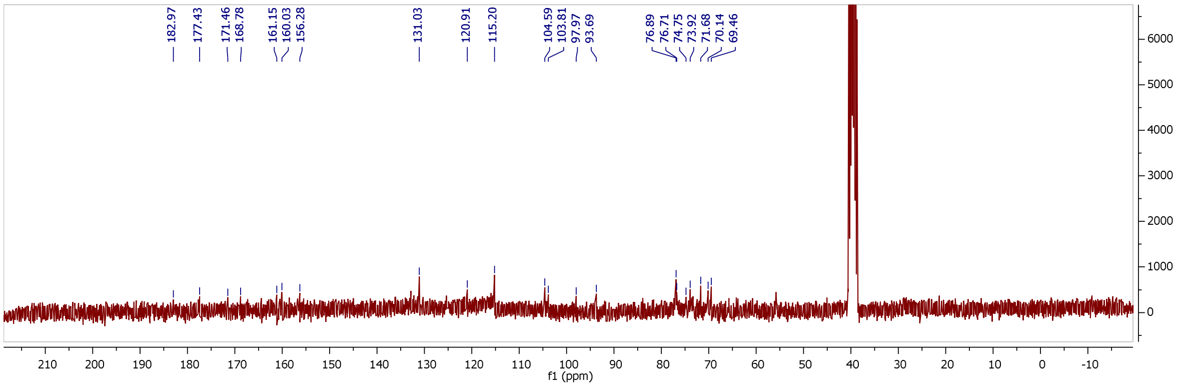

Figure S8b. ^{13}C spectrum of kaempferol-3-O-[β-xylosyl(1‴→2″)]-β-glucuronoside (**13**); 75MHz, in DMSO-d_6).

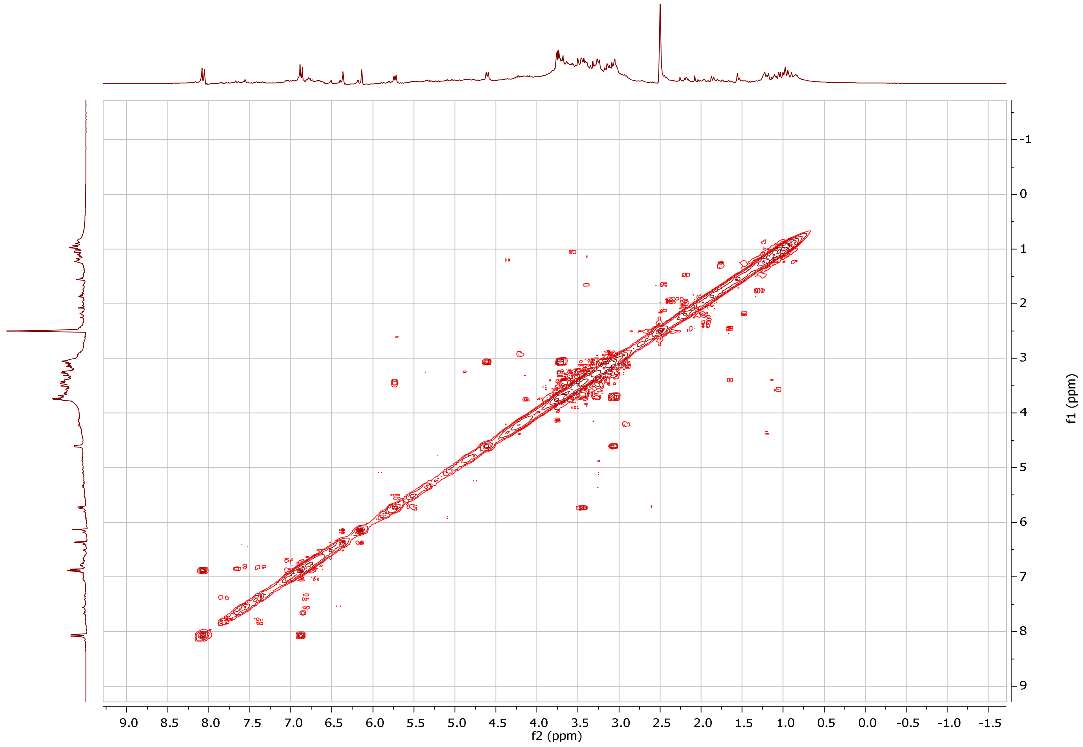

Figure S8c. ^{1}H-^{1}H COSY spectrum of kaempferol-3-O-[β-xylosyl(1‴→2″)]-β-glucuronoside (**13**); 300MHz, in DMSO-d_6).

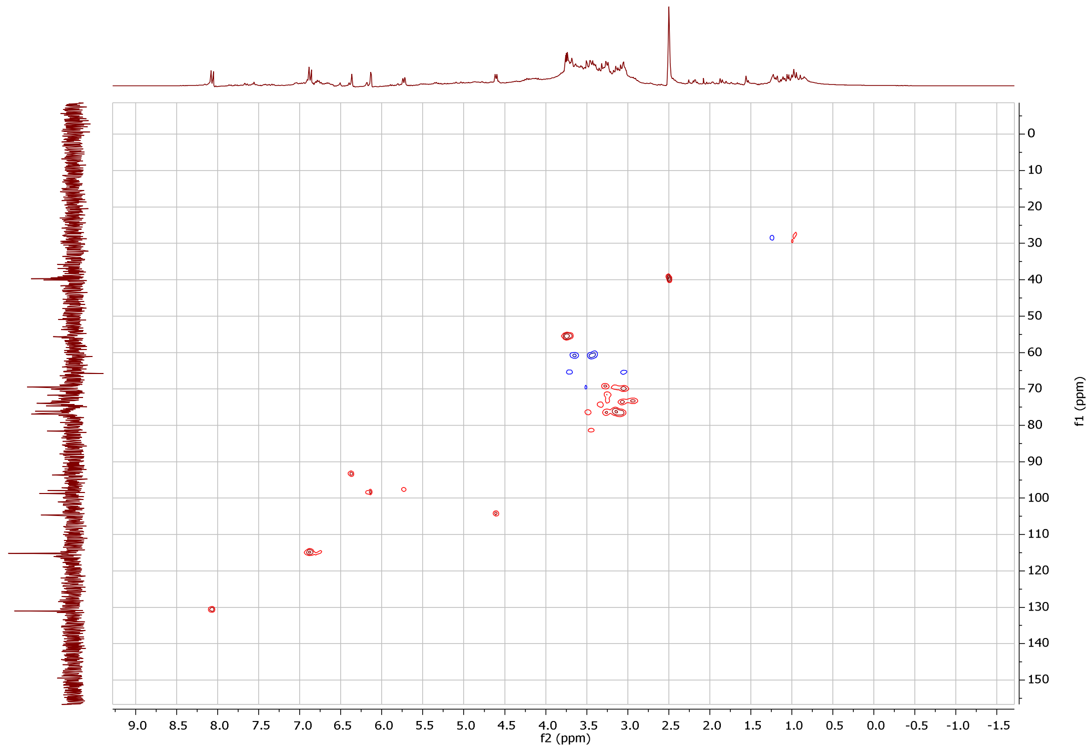

Figure S8d. ^{1}H-^{13}C HSQC spectrum of kaempferol-3-O-[β-xylosyl(1‴→2″)]-β-glucuronoside (**13**) (DMSO-d_6).

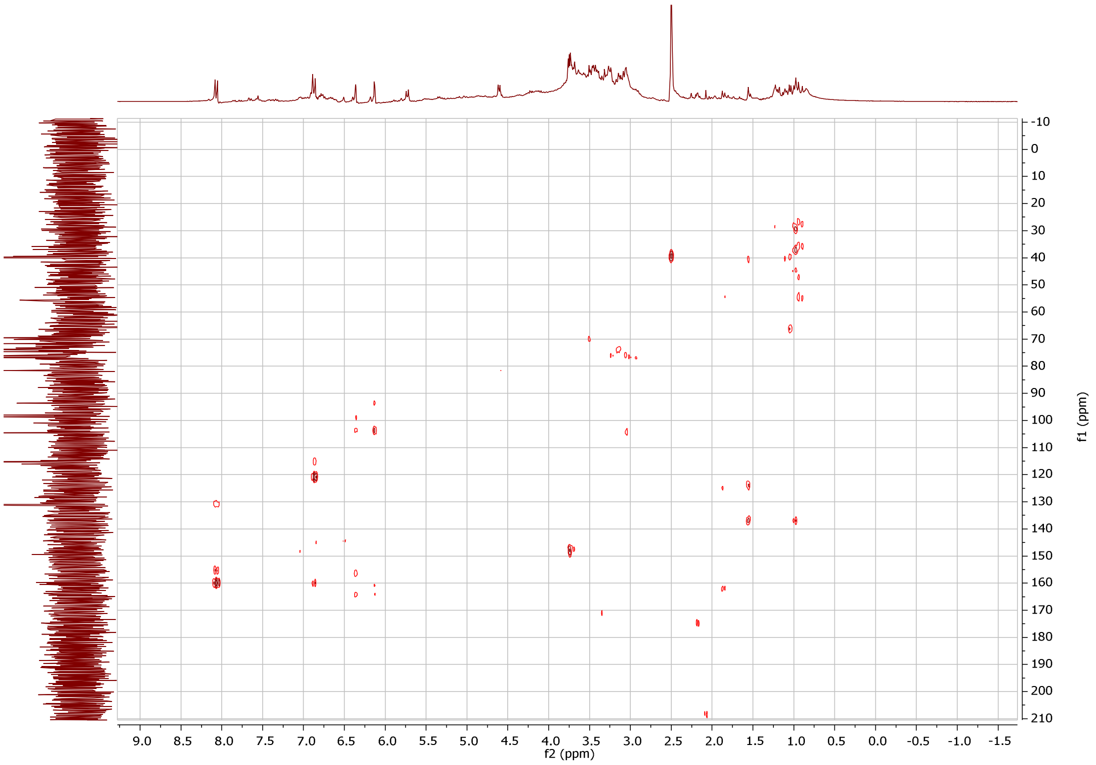

Figure S8e. ^{1}H-^{13}C HMBC spectrum of kaempferol-3-O-[β-xylosyl(1‴→2″)]-β-glucuronoside (**13**) (DMSO-d_6).

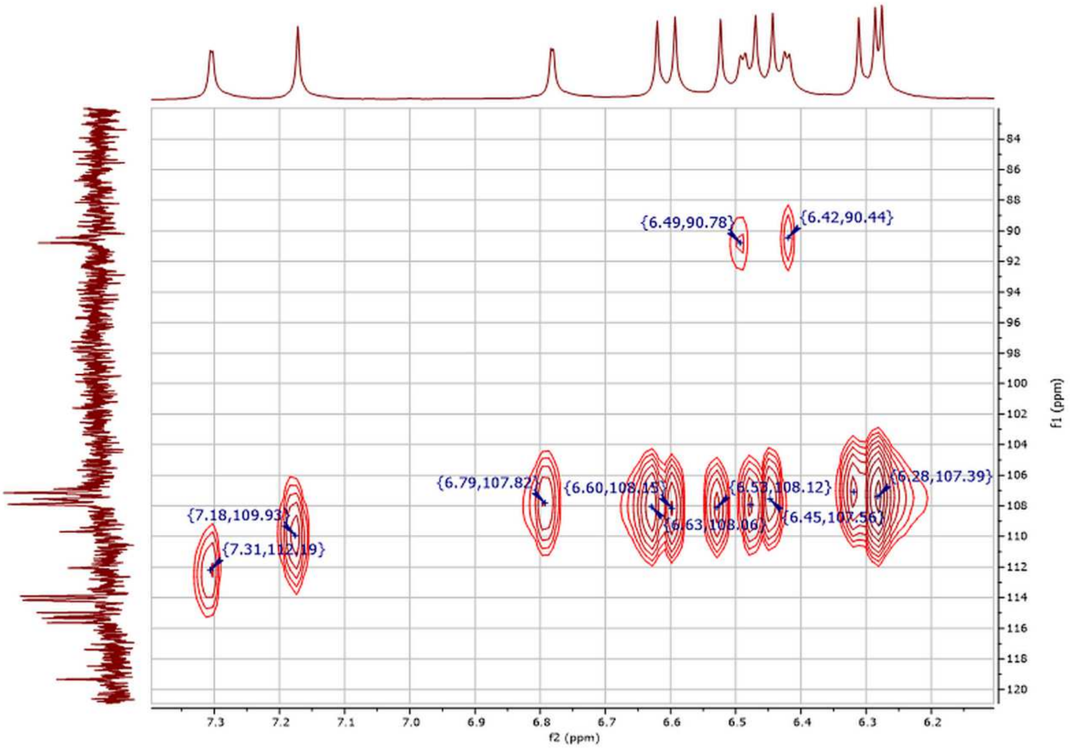

Figure S9. A detail of aromatic region of ^{1}H-^{13}C HSQC spectrum of agrimoniin (**10**); 500MHz, in equivolumetric mixture of acetone-d_6 and D_2O)

Table S1. The antiglycation activity of selected flavonols and known inhibitors (n = 3). *Abbreviations:* **Ag**, aminoguanidine (reference); **M**, metformin (reference); **Q**, quercetin (reference); **Q3g**, quercetin-3-*O*-β-glucoside (*isoquercitrin*; minor FaSS extracts constituent); **Q4′g**, quercetin-4′-*O*-β-glucoside (*spireoside*; reference); **Q3ga**, quercetin-3-*O*-β-galactoside (*hyperoside*; reference); **Q3gr**, quercetin-3-*O*-β-glucuronoside (*miquelianin*; **12**); **Q3grx**, quercetin-3-*O*-[β-xylosyl(1‴→2″)]-β-glucuronoside (*flagarin*; **11**).

Compound	% inhibition	SD	ANOVA
Ag	**74.81**	*2.16*	a
M	52.28	*13.82*	bd
Q	**77.16**	*10.05*	a
Q3g	**74.91**	*1.64*	a
Q4′g	63.93	*3.58*	c
Q3ga	**77.97**	*0.75*	a
Q3gr (**12**)	**71.56**	*5.80*	abc
Q3grx (**11**)	44.82	*3.23*	d

Values not sharing a common letter are significantly different at $P < 0.05$ by Tukey's multiple comparisons test.
